# Postdevelopmental knockout of Orai1 improves muscle pathology in a mouse model of Duchenne muscular dystrophy

**DOI:** 10.1085/jgp.202213081

**Published:** 2022-08-08

**Authors:** Maricela García-Castañeda, Antonio Michelucci, Nan Zhao, Sundeep Malik, Robert T. Dirksen

**Affiliations:** 1 Department of Pharmacology and Physiology, University of Rochester School of Medicine and Dentistry, Rochester, NY; 2 Department of Chemistry, Biology and Biotechnology, University of Perugia, Perugia, Italy.

## Abstract

Duchenne muscular dystrophy (DMD), an X-linked disorder caused by loss-of-function mutations in the dystrophin gene, is characterized by progressive muscle degeneration and weakness. Enhanced store-operated Ca^2+^ entry (SOCE), a Ca^2+^ influx mechanism coordinated by STIM1 sensors of luminal Ca^2+^ within the sarcoplasmic reticulum (SR) and Ca^2+^-permeable Orai1 channels in the sarcolemma, is proposed to contribute to Ca^2+^-mediated muscle damage in DMD. To directly determine the impact of Orai1-dependent SOCE on the dystrophic phenotype, we crossed *mdx* mice with tamoxifen-inducible, muscle-specific Orai1 knockout mice (*mdx*-Orai1 KO mice). Both constitutive and SOCE were significantly increased in flexor digitorum brevis fibers from *mdx* mice, while SOCE was absent in fibers from both Orai1 KO and *mdx*-Orai1 KO mice. Compared with WT mice, fibers from *mdx* mice exhibited (1) increased resting myoplasmic Ca^2+^ levels, (2) reduced total releasable Ca^2+^ store content, and (3) a prolonged rate of electrically evoked Ca^2+^ transient decay. These effects were partially normalized in fibers from *mdx*-Orai1 KO mice. Intact extensor digitorum longus muscles from *mdx* mice exhibited a significant reduction of maximal specific force, which was rescued in muscles from *mdx*-Orai1 KO mice. Finally, during exposure to consecutive eccentric contractions, muscles from *mdx* mice displayed a more pronounced decline in specific force compared with that of WT mice, which was also significantly attenuated by Orai1 ablation. Together, these results indicate that enhanced Orai1-dependent SOCE exacerbates the dystrophic phenotype and that Orai1 deficiency improves muscle pathology by both normalizing Ca^2+^ homeostasis and promoting sarcolemmal integrity/stability.

## Teaser

Postdevelopmental, muscle-specific ablation of Orai1 in *mdx* mice abolishes excessive constitutive and store-operated Ca^2+^ entry, improves muscular dystrophy pathology, and promotes sarcolemmal integrity, thus demonstrating an important role of enhanced Orai1-mediated Ca^2+^ entry in exacerbating the dystrophic phenotype.

## Introduction

Duchenne muscular dystrophy (DMD), an X-linked recessive muscle disorder that appears early during childhood, is characterized by muscle weakness, reduced contractile function, and progressive muscle degeneration. Despite significant advances in understanding DMD pathogenesis and the development of promising therapeutic strategies, there is currently no cure for DMD, and treatment remains primarily supportive in nature. DMD is linked to loss-of-function mutations in the gene that encodes dystrophin, a 427-kD structural protein on the myoplasmic face of the sarcolemma (Monaco et al., 1986). Dystrophin anchors γ-actin filaments of the subsarcolemmal cytoskeleton to a macromolecular assembly of proteins collectively known as the dystrophin-associated protein complex (Hoffman et al., 1987), which makes further connections via laminin to the extracellular basal lamina. Thus, loss of dystrophin disrupts this structural complex that is critical for maintaining sarcolemmal integrity during mechanical stress. However, the precise mechanisms that underlie progressive muscle fiber degeneration and necrosis in DMD continue to be debated. A long-standing hypothesis for muscle fiber degeneration in DMD is that a sustained elevation in myoplasmic Ca^2+^ triggers multiple intracellular downstream pathways/mechanisms, including mitochondrial Ca^2+^ overload/damage, enhanced oxidative stress, and activation of Ca^2+^-dependent proteases that together promote myofiber degeneration and death (Durbeej and Campbell, 2002).

Currently, two main pathomechanisms are hypothesized to underlie abnormalities in myoplasmic Ca^2+^ homeostasis in DMD: (1) enhanced Ca^2+^ leak from the sarcoplasmic reticulum (SR) through oxidized type 1 ryanodine receptor (RyR1) Ca^2+^ release channels and (2) excessive extracellular Ca^2+^ influx. The first pathomechanism is supported by several studies showing that loss of dystrophin is associated with hypernitrosylation of specific cysteine residues in RyR1 leading to FKBP12 dissociation from RyR1, destabilization of the RyR1 channel closed state, and increased RyR1-dependent SR Ca^2+^ leak (Bellinger et al., 2009; Andersson et al., 2012). Abnormal SR Ca^2+^ leak subsequently leads to mitochondrial Ca^2+^ overload and uncontrolled production of reactive species of oxygen and nitrogen (ROS and RNS, respectively) that further promotes RyR1 oxidization/nitrosylation in a destructive feed-forward cycle of increased RyR1 Ca^2+^ leak and ROS/RNS production. Besides enhancing RyR1 opening probability, ROS/RNS-dependent oxidative stress can also reduce the activity of the SR/ER Ca^2+^ ATPase (SERCA), the main Ca^2+^ pump in the SR membrane responsible for Ca^2+^ reuptake (Viner et al., 1996; Sharov et al., 2006; Dremina et al., 2007; Qaisar et al., 2021). Indeed, a reduction of SERCA activity due to increased oxidative stress was reported previously in dystrophic muscles (Divet and Huchet-Cadiou, 2002; Kargacin and Kargacin, 1996). The importance of increased oxidative stress as a key pathomechanism of muscle degeneration in DMD is further supported by improvement of the dystrophic phenotype following treatment with *N*-acetylcysteine, a potent antioxidant (Whitehead et al., 2008).

The second pathomechanism for altered Ca^2+^ homeostasis in DMD involves a net increase in trans-sarcolemmal Ca^2+^ influx through “microtears” and/or Ca^2+^-permeable ion channels (e.g., leak, stretch-activated, receptor-activated, and store-operated channels; Boittin et al., 2006; Fong et al., 1990; Franco and Lansman, 1990; Vandebrouck et al., 2002; Millay et al., 2009). A growing number of studies provide evidence for a modulatory role of enhanced store-operated Ca^2+^ entry (SOCE) in the pathogenesis of DMD (Edwards et al., 2010; Zhao et al., 2012; Cully et al., 2012). SOCE is a ubiquitous Ca^2+^ influx mechanism triggered by the depletion of intracellular Ca^2+^ stores, that allows for the recovery of Ca^2+^ ions from the extracellular space needed to both refill ER/SR and activate downstream Ca^2+^ signaling pathways (e.g., NFAT). The two proteins that form the core SOCE machinery are (1) stromal interaction molecule-1 (STIM1), which senses changes in luminal ER/SR Ca^2+^ levels (Roos et al., 2005; Zhang et al., 2005; Liou et al., 2005), and (2) Orai1, the Ca^2+^ release-activated Ca^2+^ channel in the surface membrane (Feske et al., 2006; Yeromin et al., 2006; Vig et al., 2006). STIM1 and Orai1 also coordinate SOCE in skeletal muscle (Stiber et al., 2008; Lyfenko and Dirksen, 2008; Dirksen, 2009). Consistent with the idea that dysregulated activation of STIM1/Orai1-dependent SOCE promotes Ca^2+^-mediated muscle fiber degeneration in muscular dystrophy, STIM1 and Orai1 expression levels and SOCE activity were reported to be increased in dystrophin-deficient *mdx* mice (Edwards et al., 2010; Zhao et al., 2012; Cully et al., 2012). In addition, early developmental inhibition of SOCE function significantly mitigates the dystrophic phenotype observed in both dystrophin-null (*mdx*) and γ-sarcoglycan–null mice (Goonasekera et al., 2014).

Because enhanced STIM1/Orai1-mediated SOCE is proposed to be an important mechanism of aberrant Ca^2+^ influx in *mdx* mice, we evaluated the role of Orai1-dependent Ca^2+^ entry in the DMD phenotype by crossing *mdx* mice with muscle-specific, tamoxifen-inducible, Orai1-knockout (Orai1 KO) mice. The resulting compound offspring enable tamoxifen-inducible ablation of Orai1 in adult *mdx* mice (*mdx*-Orai1 KO mice). We found that ablation of Orai1 in young adult (2–3-mo-old) *mdx* mice abolished Orai1-dependent Ca^2+^ entry (e.g., SOCE), normalized intracellular Ca^2+^ homeostasis, improved muscle performance, and unexpectedly protected muscles from eccentric contraction–induced damage. Furthermore, postdevelopmental Orai1 KO normalized fiber cross-sectional area (CSA) and reduced muscle fibrosis, a marker of muscle damage and necrosis. These results demonstrate an important role of enhanced Orai1-mediated Ca^2+^ entry in exacerbating the dystrophic phenotype of *mdx* mice, rendering Orai1 an attractive potential therapeutic target for the treatment of DMD.

## Materials and methods

### Mice

**Figure 1.**
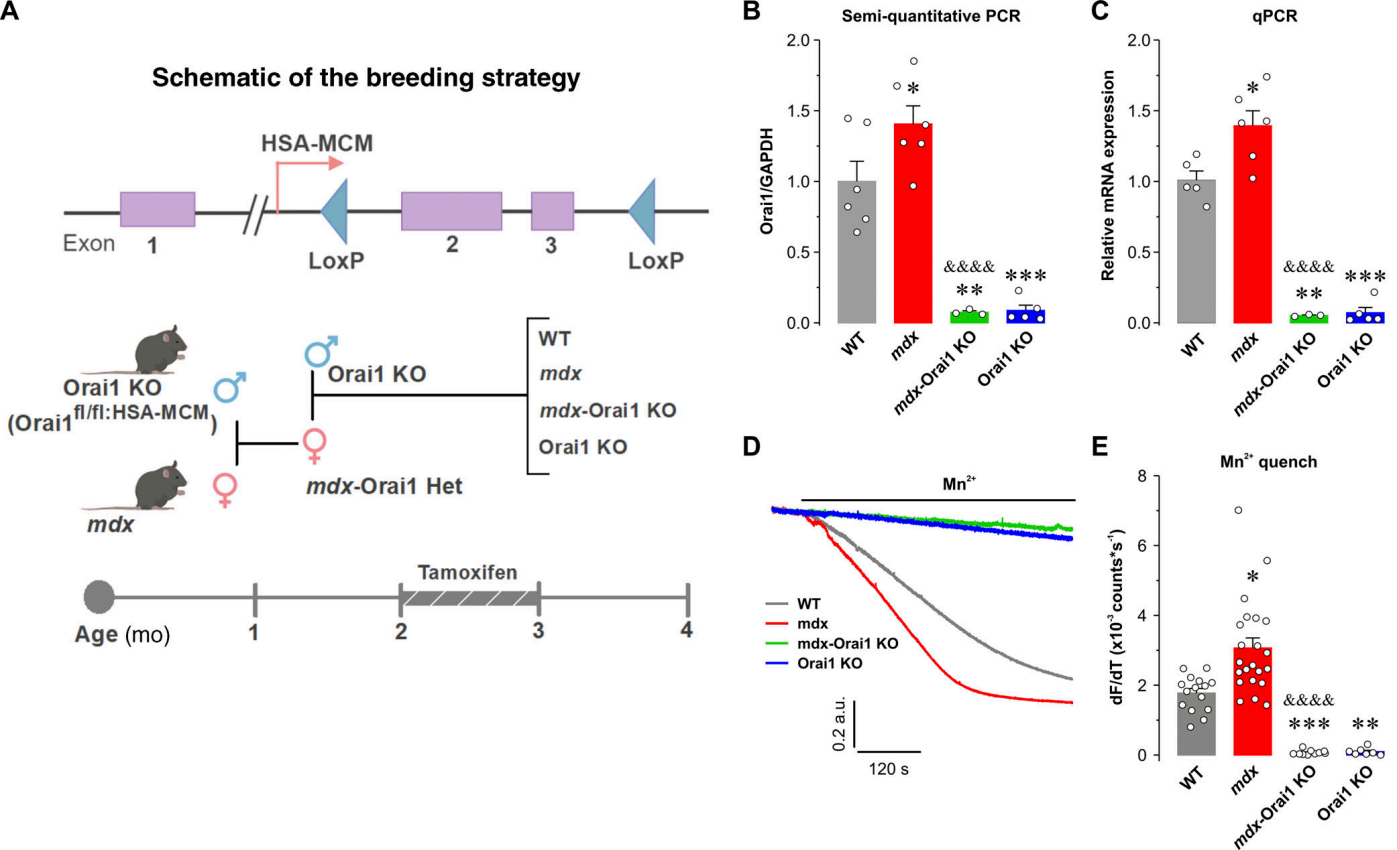
**Generation and validation of *mdx-*Orai1 KO mice. (A)** Schematic of the breeding strategy used to generate muscle-specific, tamoxifen-inducible *mdx*-Orai1 KO mice. Top: Diagram of LoxP sites within the Orai1 gene (Carrell et al., 2016). Center: Diagram of a male muscle-specific, tamoxifen-inducible Orai1 KO (HSA-MCM-Orai1^*fl/fl*^) mouse crossed with a homozygous female *mdx* mouse. The dystrophin-heterozygous female F1 generation mouse with a single Orai1 KO allele (*mdx-*HSA-MCM-Orai1^*fl/+*^) was crossed with a male Orai1 KO mouse to obtain the following four genotypes: WT, *mdx*, *mdx*-Orai1 KO, and Orai1 KO mice. Bottom: Tamoxifen treatment paradigm: all 2-mo-old male mice were treated for 1 mo with a tamoxifen-supplemented diet. Experiments were initiated 1 wk after the treatment ended. The breeding scheme was created using BioRender.com. **(B)** Bar graph summarizing semiquantitative RT-PCR measurements of Orai1 transcript levels normalized to GAPDH transcript in WT (*n* = 6), *mdx* (*n* = 6), *mdx*-Orai1 KO (*n* = 3), and Orai1 KO (*n* = 5) mice. **(C)** Bar graph summarizing quantitative RT-PCR measurements of Orai1 transcript levels normalized to GAPDH transcript in WT (*n* = 5), *mdx* (*n* = 6), *mdx*-Orai1 KO (*n* = 3), and Orai1 KO (*n* = 5) mice. **(D)** Representative superimposed traces of Mn^2+^ quench of fura-2 fluorescence recorded from single FDB fibers after prior treatment for 1 h in a Ca^2+^-free solution supplemented with 1 µM TG and 15 µM CPA to fully deplete SR Ca^2+^ stores. **(E)** Bar graph summarizing the maximum rate (counts/s) of Mn^2+^ quench in fibers from WT (*n* = 15 fibers), *mdx* (*n* = 22 fibers), *mdx*-Orai1 KO (*n* = 9 fibers), and Orai1 KO (*n* = 7 fibers) mice. Data are shown as mean ± SEM. For B, *, P = 0.0470; **, P = 0.0005; ***, P = 0.0001 for *mdx*, *mdx*-Orai1 KO, and Orai1 KO mice, respectively, relative to WT; ^&&&&^, P = 0.000015, relative to *mdx*. For C, *, P = 0.0457; **, P = 0.00052; ***, P = 0.0002 for *mdx*, *mdx*-Orai1 KO, and Orai1 KO mice, respectively, relative to WT; ^&&&&^, P = 0.000000084, relative to *mdx*. For E, *, P = 0.046; ***, P = 0.006; **, P = 0.0253 for *mdx*, *mdx*-Orai1 KO, and Orai1 KO mice, respectively, relative to WT; ^&&&&^, P = 0.00000016, relative to *mdx* by one-way ANOVA followed by post hoc Dunnett’s test. Number of mice used in E: WT (*n* = 5), *mdx* (*n* = 6), *mdx*-Orai1 KO (*n* = 3), and Orai1 KO (*n* = 4).

Tamoxifen-inducible, muscle-specific Orai1-KO (HSA-MCM Orai1^*fl/fl*^) mice were generated as previously described (Carrell et al., 2016). Briefly, we generated dystrophic mice that permit inducible Orai1 ablation in adult skeletal muscle by crossing male HSA-MCM Orai1^*fl/fl*^ mice with homozygous female *mdx* mice (C57Bl/10ScSn-DMDmdxIJ; Jackson Laboratories). This strategy enabled generation of F1 heterozygous female *mdx* mice carrying a single floxed Orai1 allele (*mdx*-Orai1 Het). These mice were then mated with male HSA-MCM Orai1^*fl/fl*^ mice to obtain dystrophin-deficient mice carrying two floxed Orai1 alleles (*mdx*-Orai1 KO). Using this breeding strategy (see Fig. 1 A), we obtained the following four genotypes used in this study: (1) WT mice (HSA-MCM negative Orai1^*fl/fl*^ or Orai1^*+/fl*^), (2) *mdx* mice (*mdx-*HSA-MCM negative Orai1^*fl/fl*^ or Orai1^*+/fl*^), (3) Orai1 KO mice (HSA-MCM-Orai1^*fl/fl*^) mice, and (4) *mdx*-Orai1 KO mice (*mdx-*HSA-MCM-Orai1^*fl/fl*^). The *mdx* allele was detected using the following primers: (forward) 5′-AAC​TCA​TCA​AAT​ATG​CGT​GTT​AGT-3′; (reverse) 5′-GCC​CCT​CAA​TCT​CTT​CAA​ATT​C-3′. The MerCreMer and Orai1 floxed alleles were verified according to protocols described previously (Carrell et al., 2016). To induce Orai1 KO, 2-mo-old mice were treated with a tamoxifen-supplemented diet (Envigo; TD.130860) for 4 wk (while monitoring normal weight gain). Mice were then fed regular mouse chow for ≥1 wk. As DMD is an X-linked disease, all the experiments were restricted to 13–16-wk-old male mice. All animal studies were designed to minimize animal suffering and were approved by the University Committee on Animal Resources at the University of Rochester (UCAR2006-114E).

### Treadmill endurance running and grip strength

3–4-mo-old mice were pretrained on a 6-lane treadmill (Columbus Instruments) at a modest treadmill speed of 5 m/min for 5 min at a 15° incline over 3 consecutive days. On the fourth day, mice were subjected to a 1-h endurance run on the treadmill (1 km of total distance covered), starting at 5 m/min for 5 min, followed by increments of 1 m/min until reaching a maximum speed of 20 m/min, and then maintaining this speed for 40 min. Continued running was encouraged by delivering brief (<1 s) spurts of air on the mouse’s backside using a Whoosh Duster. The number of rests during each 5-min window of time was recorded for each mouse. Exhaustion was defined as an inability of the mouse to re-engage the treadmill after three consecutive, <1-s spurts of air as described previously (Carrell et al., 2016). The number of rests and the total distance run were recorded for each mouse. In vivo mouse forelimb grip strength was assessed using a digital force gauge (GTX; Dillon) as described previously (Loy et al., 2011).

### Semiquantitative RT-PCR

Semiquantitative RT-PCR was used to assess relative changes in Orai1 transcript as previously reported (Carrell et al., 2016). Briefly, total RNA was isolated from tibialis anterior (TA) muscles using TRIzol (cat. 15596-018; Thermo Fisher Scientific) and quantified. RNA (1 μg) was then treated with DNase according to the manufacturer’s protocol (EN0525; Thermo Fisher Scientific) and reverse transcribed using a Super Script III First-Strand Synthesis System primed with oligonucleotides (dT; cat. 18080-051; Thermo Fisher Scientific). Semiquantitative PCR was carried out on 10 ng cDNA using 5′-end fluorescein (6-FAM)–labeled forward primers (Integrated DNA Technologies). Reactions were quantified every 2 cycles from 22 to 32 cycles to verify that amplification of both control *GAPDH* and *Orai1* cDNAs were within the linear range. Primers used were mouse *GAPDH* (921 bp): (forward) 5′-AGG​CCG​GTG​CTG​AGT​ATG​TC-3′; (reverse) 5′-GGG​TGC​AGC​GAA​CTT​TAT​TGA​TGG-3′; and mouse *Orai1* (307 bp): (forward) 5′-TTT​AGT​GCC​TGC​ACC​ACA​GTG​CTA-3′; (reverse) 5′-TGT​GGT​TGG​CGA​CGA​TGA​CTG​ATT-3′.

### Quantitative RT-PCR

Orai1 transcript level was also assessed using a quantitative RT-PCR approach on RNA isolated from TA muscles as described above. Quantitative PCR was performed with 10 ng cDNA on a StepOnePlus Real-Time PCR machine (Applied Biosystems) using SYBR Green FastMix (Quantabio). Relative mRNA transcript levels from each experiment were performed in triplicate and standardized to their own internal *GAPDH* gene expression, then to the control condition using the 2^−∆∆CT^ analysis method, where CT is count threshold, in accordance with Minimum Information for Publication of Quantitative Real-Time PCR Experiments (MIQE) guidelines (Bustin et al, 2009). Primers used were mouse *Orai1* (forward): 5′-GAT​CGG​CCA​GAG​TTA​CTC​CG-3′, (reverse): 5′-TGG​GTA​GTC​ATG​GTC​TGT​GTC-3′; and mouse *GAPDH* (forward): 5′-AGG​TCG​GTG​TGA​ACG​GAT​TTG-3′, (reverse): 5′-GGG​GTC​GTT​GAT​GGC​AAC​A-3′.

### Isolation of single muscle fibers

Flexor digitorum brevis (FDB) muscles were dissected from mouse hind paws and placed in a dish containing Ringer’s solution consisting of 145 mM NaCl, 5 mM KCl, 2 mM CaCl_2_, 1 mM MgCl_2_, and 10 mM HEPES, pH 7.4. Muscles were then incubated in Ringer’s solution supplemented with 1 mg/ml collagenase A for 60 min while rocking gently at 37°C to allow for enzymatic dissociation. Single FDB fibers obtained by mechanical dissociation/trituration were plated on glass-bottom dishes and allowed to settle for ≥20 min before conducting single-fiber experiments (detailed below). Only fibers with a clean morphology, clear striations, and no signs of swelling or damage were selected for experiments.

### Mn^2+^ quench of fura-2 fluorescence

For Mn^2+^ quench studies, isolated FDB fibers were loaded with 5 μM fura-2 AM for 1 h at 37°C in a Ca^2+^-free Ringer’s solution containing 145 mM NaCl, 5 mM KCl, 3 mM MgCl_2_, and 0.2 mM EGTA, pH 7.4. During fura-2 loading, fibers were also incubated with two SERCA pump inhibitors (1 μM thapsigargin [TG] and 15 μM cyclopiazonic acid [CPA]) to fully deplete SR Ca^2+^ stores before measurement of the maximal rate of store-dependent Mn^2+^ quench (store-dependent entry or SOCE). In a second set of experiments, FDB fibers were loaded with fura-2 AM in the absence of SERCA pump inhibitors (store-independent entry or constitutive entry). To prevent movement artifacts during Mn^2+^ quench recordings, 30 μM *N*-benzyl-*p*-toluene sulfonamide (BTS) was also included in the extracellular solution (Wei-Lapierre et al., 2013; Michelucci et al., 2019). Fibers were then rinsed with Ca^2+^-free Ringer solution and excited at 362 nm (isosbestic point of fura-2), while emission was detected at 510 nm using a DeltaRam illumination system (Photon Technology International). After obtaining an initial baseline rate of fura-2 decay (*R*_baseline_), fibers were exposed to Ca^2+^-free Ringer’s supplemented with 0.5 mM MnCl_2_. The maximum rate of change in fura-2 fluorescence in the presence of Mn^2+^ (*R*_max_) was obtained from the peak time derivative of the fura-2 emission trace during Mn^2+^ application. The maximum rate of entry (*R*_ENTRY_; either with or without prior store depletion) was then calculated as *R*_ENTRY_ = *R*_max_ − *R*_baseline_ and expressed as d*F*/d*t* in counts/s as described previously (Wei-Lapierre et al., 2013; Michelucci et al., 2019).

### Electrically evoked Ca^2+^ transients

Myoplasmic Ca^2+^ transients were quantified in single FDB fibers loaded at room temperature for 20 min with 4 µM mag-fluo-4 AM, a rapid, low-affinity Ca^2+^ dye that enables resolution of the magnitude and kinetics of electrically evoked Ca^2+^ transients (Capote et al., 2005), followed by washout in dye-free solution supplemented with 25 µM BTS for 20 min. FDB fibers were then plated on glass-bottom dishes and mounted on the stage of a Nikon Eclipse 2000E inverted microscope. Twitch electrical stimulations were delivered to individual FDB fibers using a 150 mM NaCl-filled glass electrode placed adjacent to the cell of interest. Fibers were electrically stimulated with five consecutive twitch (0.5-Hz) stimulations. Mag-fluo-4 was excited at 480 ± 15 nm using an Excite epifluorescence illumination system (Nikon Instruments) with fluorescence emission collected at 535 ± 30 nm using a 40× oil objective and photomultiplier detection system (Photon Technologies). Relative changes in mag-fluo-4 fluorescence from baseline (*F*/*F*_0_) were digitized at 10 kHz and analyzed using Clampfit 10.0 (Molecular Devices). The peak change in relative mag-fluo-4 fluorescence (Δ*F*/*F*) was calculated as the peak fluorescence divided by the baseline minus 1 (García and Beam, 1994). The decay phase of each transient was fitted according to the following second order exponential equation:

$$F\left(t\right)=A_{fast}\times \left[\exp\left(-t/\tau_{fast}\right)\right]+A_{slow}\times \left[\exp\left(-t/\tau_{slow}\right)\right],$$

where *F*(*t*) is the fluorescence at time *t*, *A*_*fast*_ and τ_*fast*_ are the amplitude and time constants of the fast component, respectively, and *A*_*slow*_ and τ_*slow*_ are the amplitude and time constants of the slow component, respectively (Capote et al., 2005).

### Total releasable Ca^2+^ store content

Total releasable Ca^2+^ store content was determined from single FDB fibers loaded with 4 µM fura-FF AM, a low-affinity, ratiometric Ca^2+^ dye, for 30 min at room temperature in control Ringer’s solution followed by 30-min incubation in dye-free Ringer’s solution supplemented with 40 µM BTS, as described previously (Zvaritch et al., 2007; Loy et al., 2011; Wei-Lapierre et al., 2013; Linsley et al., 2017; Michelucci et al., 2019; Michelucci et al., 2020). Fibers were then perfused in Ca^2+^-free Ringer’s solution while alternately excited at 340 and 380 nm (510-nm emission) every 250 ms (30-ms exposure per wavelength and 2 × 2 binning) using a monochromator-based illumination system (TILL Photonics). Fura-FF emission at 535 ± 30 nm was captured using a high-speed, digital QE charge-coupled device camera (TILL Photonics). Total releasable Ca^2+^ store content was assessed from the difference between baseline and peak fura-FF ratios (ΔRatio_340/380_) upon application of a Ca^2+^ store release cocktail containing 10 µM ionomycin, 30 µM CPA, and 100 µM EGTA in a Ca^2+^-free Ringer’s solution (ICE) as previously described (Loy et al., 2011; Wei-Lapierre et al., 2013; Michelucci et al., 2019, Michelucci et al., 2020). To confirm that the peak of the fura-FF signal during ICE application was not saturated, maximal fura-FF responsiveness was assessed at the end of each experiment by subsequent application of normal Ca^2+^-containing Ringer’s solution. Analysis of the peak ICE-induced change in fura-FF ratio (ΔRatio_340/380_) was calculated using Clampfit 10.0 (Molecular Devices).

### Resting myoplasmic Ca^2+^ concentration

Single FDB fibers were loaded with 5 µM fura-2 AM for 30 min at room temperature in control Ringer’s solution followed by a 30-min incubation with dye-free Ringer’s solution. Fura-2–loaded fibers were then placed on the stage of an inverted epifluorescence microscope (Nikon Instruments) and alternately excited at 340 and 380 nm (30-ms exposure per wavelength and 2 × 2 binning) using a monochromator-based illumination system. The fluorescence emission at 510 nm was captured using a high-speed, digital QE charge-coupled device camera (TILL Photonics). Fura-2 340/380 ratios from myoplasmic areas of interest were calculated using TILLvisION software, analyzed offline using ImageJ, and then converted to resting free Ca^2+^ concentrations using an in situ calibration curve for fura-2 generated as described previously (Michelucci et al., 2019).

### Ex vivo contractility and eccentric muscle damage

Ex vivo assessments of muscle-specific force, fatigue during repetitive high-frequency stimulation, and eccentric muscle damage were made in excised extensor digitorum longus (EDL). Muscle-specific force and eccentric muscle damage were also assessed in soleus muscles. Briefly, mice were anesthetized by intraperitoneal injection of an anesthetic cocktail as described previously (Wei-Lapierre et al., 2013). EDL and soleus muscles were isolated, tied using 4-0 surgical suture, carefully excised, attached to a servo motor and force transducer (1200 A, Aurora Scientific), and placed between two platinum electrode plates in a chamber continuously perfused with oxygenated Ringer solution containing 137 mM NaCl, 5 mM KCl, 1.2 mM NaH_2_PO_4_, 1 mM MgSO_4_, 2 mM CaCl_2_, 10 mM glucose, and 24 mM NaHCO_3,_ pH 7.4. Before starting each experiment, optimal stimulation intensity and muscle length (*L*_*o*_) were determined using a series of 1-Hz twitch stimulation trains to guide stretching the muscle to the length that generated maximal force (*F*_*o*_). After establishing *L*_*o*_, muscles were first equilibrated using three 500-ms, 150-Hz tetani delivered at 1-min intervals. EDL and soleus muscles were then subjected to a force–frequency stimulation protocol (from 1 to 250 Hz for EDL muscles; from 1 to 200 Hz for soleus muscles). To assess muscle fatigability, EDL muscles were subjected to a repetitive, high-frequency stimulation protocol (40 stimulus trains of 50 Hz and 500 ms in duration delivered every 2.5 s). To assess susceptibility to damage during eccentric contractions, a different set of EDL and soleus muscles were subjected to a repetitive eccentric contraction protocol optimized for eccentric contraction–induced damage as previously described (Lindsay et al., 2020). This protocol consisted of 10 successive 700-ms, 150-Hz eccentric contractions (500 ms of isometric contraction followed by an additional 200 ms during a 10% of stretch of *L*_*o*_ at a speed of 0.5 *L*_*o*_/s). Muscle physiological CSA and specific force were calculated as described previously (Hakim et al., 2011).

### Immunostaining and CSA analysis

TA muscles were excised and incubated in 30% sucrose overnight at 4°C (Bachman et al., 2018). Muscles were mounted in optimal cutting temperature medium and snap frozen in liquid nitrogen–cooled 2-methylbutane (Carrell et al., 2016). TA muscle cryosections (10 μm thick) were fixed in 4% paraformaldehyde (3–5 min), washed with PBS, and permeabilized by exposure to 0.1% Triton X-100 in PBS for 5 min. Sections were washed again with PBS, blocked with 3% BSA for 30 min at room temperature, and incubated for 2 h at room temperature with anti-laminin antibody (rat monoclonal diluted 1:100; Sigma-Aldrich). Sections were then washed with PBS and incubated with Alexa Fluor 488 (anti-rat polyclonal; 1:400 diluted; Invitrogen) for 1 h at room temperature. Nuclei were stained with Hoechst dye (0.2 μg/μl for 6 min at room temperature) and washed with PBS before being mounted with VectaShield (Vector Laboratories) for analysis by confocal microscopy. Fiber boundaries were defined by the laminin signal, and CSA was calculated using Fiji software as described previously (Tyagi et al., 2017). To examine gross muscle structure and number of centrally nucleated fibers, cryosections were stained with hematoxylin and eosin as described previously (Brennan et al., 2019).

### Hydroxyproline content

Diaphragm muscles were excised, carefully cleaned to remove attached tendons, weighed, and snap frozen in liquid nitrogen. Collagen content was measured using a hydroxyproline assay kit (MAK008; Sigma-Aldrich). Briefly, diaphragm muscles were homogenized in water and hydrolyzed overnight in 6 M hydrochloric acid at 110°C. 10 µl of homogenate was then transferred to a 96-well plate and incubated at 60°C to dry the sample. Dried samples were resuspended in chloramine T/oxidation buffer mixture, incubated at room temperature for 5 min, mixed with DMAB-perchloric acid/isopropanol reagent, and then incubated for 90 min at 60°C. Sample absorbance was measured at 560 nm using a FlexStation 3 Multi-Mode Microplate Reader (Molecular Devices). Absorbances (ODs) obtained for each sample were referred against a standard curve to quantify the amount of hydroxyproline, which was then normalized to milligrams of tissue wet-weight.

### Western blot analyses

EDL muscles were dissected, snap frozen in liquid nitrogen, and homogenized in RIPA lysis buffer (20 mM Tris-HCl, 150 mM NaCl, 1 mM Na_2_EDTA, 1 mM EGTA, 1% NP-40, 1% sodium deoxycholate, 2.5 mM sodium pyrophosphate, 1 mM β-glycerophosphate, 1 mM Na_3_VO_4_, and 1 μg/ml leupeptin, pH 7.5) supplemented with a cocktail of protease inhibitors. Protein concentration was determined spectrophotometrically using a Lowry method. Briefly, 10 μg of total protein samples were loaded in 10–12% SDS-PAGE polyacrylamide gel, transferred to nitrocellulose membranes, and blocked with 3% BSA in Tris-buffered saline 0.1% and Tween 20 (TBS-T) overnight. Membranes were probed with the following primary antibodies diluted with 1% BSA in TBS-T for 2 h at room temperature: dystrophin (rabbit polyclonal diluted 1:1,000; Abcam), utrophin (mouse monoclonal diluted 1:1,000; Santa Cruz Biotechnology), STIM1 (rabbit polyclonal diluted 1:5,000; Sigma-Aldrich), SERCA (rabbit polyclonal 1:10,000; Santa Cruz Biotechnology), CASQ1 (mouse monoclonal diluted 1:5,000; Thermo Fisher Scientific), β-tubulin (mouse monoclonal diluted 1:2,000; Invitrogen), and GAPDH (mouse monoclonal diluted 1:50,000; Invitrogen). β-Tubulin was used as a loading control for dystrophin and utrophin, whereas GAPDH was used as a loading control for STIM1, SERCA, and CASQ1. Membranes were then washed three times in TBS-T and incubated with either goat anti-mouse IgG-800 or goat anti-rabbit IgG-800 (both diluted 1:10,000; Invitrogen) secondary antibodies diluted in 1% BSA TBS-T for 1 h at room temperature. Proteins were visualized with an Odyssey Infrared imager from Li-Cor. Densitometry analysis and quantification were performed on exported TIFF images using ImageJ (National Institutes of Health).

### Statistical analyses

**Figure S1.**
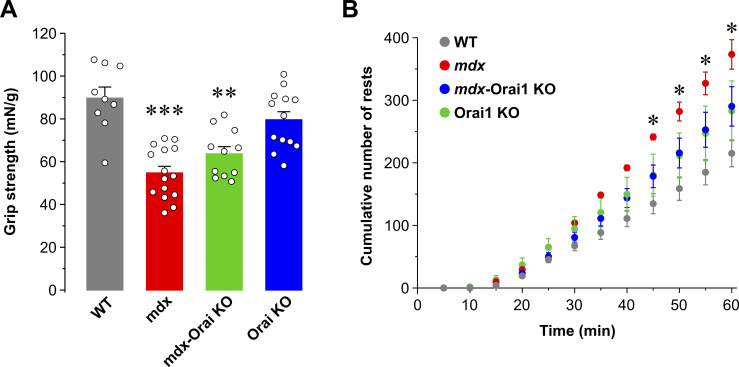
**Postdevelopmental, muscle-specific ablation of Orai1 improves muscle performance of *mdx* mice. (A)** Bar graph summarizing peak grip strength (mN) normalized to total body weight (g) assessed in WT (gray; *n* = 7 mice), *mdx* (red; *n* = 8 mice), *mdx*-Orai1 KO (green; *n* = 8 mice), and Orai1 KO (blue; *n* = 6 mice) mice. **(B)** Average cumulative number of rests during a 1-h forced treadmill endurance protocol in WT (gray; *n* = 7 mice), *mdx* (red; *n* = 8 mice), *mdx*-Orai1 KO (green; *n* = 7 mice), and Orai1 KO (blue; *n* = 10 mice) mice. Rests were defined as the number of times mice required encouragement to continue to run with using brief spurts of air using a Whoosh Duster. Data are shown as mean ± SEM; in A, ***, P = 0.00032 and **, P = 0.0074, relative to WT. In B, *, P = 0.030 at 45 min; *, P = 0.041 at 50 min; *, P = 0.040 at 55 min; *, P = 0.038 at 60 min, relative to WT by one-way ANOVA followed by post-hoc Dunnett’s test.

**Figure 2.**
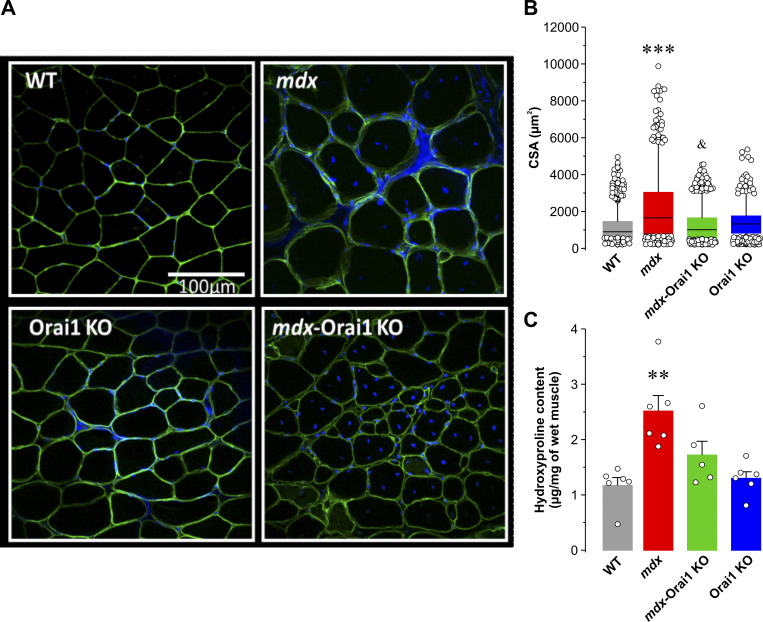
**Quantitative analyses of muscle fiber CSA and fibrosis. (A)** Representative confocal images of TA muscle transversal sections from WT, *mdx*, *mdx*-Orai1 KO, and Orai1 KO mice immunostained with anti-laminin antibody (green) to define the sarcolemma and Hoechst dye (blue) to visualize nuclei. **(B)** Bar graph summarizing average fiber CSA for each of the four genotypes shown in A: WT (*n* = 1,467 fibers), *mdx* (*n* = 741 fibers), *mdx*-Orai1 KO (*n* = 1,343 fibers), and Orai1 KO (*n* = 1,367 fibers) mice. **(C)** Hydroxyproline levels (mg/mg wet muscle weight) in diaphragm muscle homogenates from WT (*n* = 6 muscles), *mdx* (*n* = 6 muscles), *mdx*-Orai1 KO (*n* = 5 muscles), and Orai1 KO mice (*n* = 6 muscles). Data are shown as mean ± SEM. In B, ***, P = 0.00025, relative to WT; ^&^, P = 0.0038, relative to *mdx*. In C, **, P = 0.0046, relative to WT using by one-way ANOVA followed by post hoc Dunnett’s test. Number of mice used in B and C: WT (*n* = 3), *mdx* (*n* = 3), *mdx*-Orai1 KO (*n* = 3), and Orai1 KO (*n* = 3).

All data groups were verified to follow a normal distribution using an Anderson–Darling (*A*^2^) test before conducting parametric statistical analyses. The following analyses were performed blinded: (1) quantitative analyses of muscle fiber CSA and fibrosis (Fig. 2), (2) in vivo assessment of grip strength and cumulative rests during treadmill endurance protocol (Fig. S1), and (3) quantitative histologic analysis of centrally nucleated fibers (Fig. S3 B). Statistical significance was determined using one-way ANOVA followed by Dunnett’s test or ANOVA followed by post hoc Tukey test for repeated measures. A non-parametric test was used when data did not follow a normal distribution. Data were analyzed using Prism v8.0.2 for Windows (GraphPad Software), Origin 8.0 (OriginLab Corp.), pCLAMP (Molecular Devices), and ImageJ. In all cases, differences were considered statistically significant at P < 0.05. All data are presented as mean ± SEM.

### Online supplemental material

**Figure S2.**
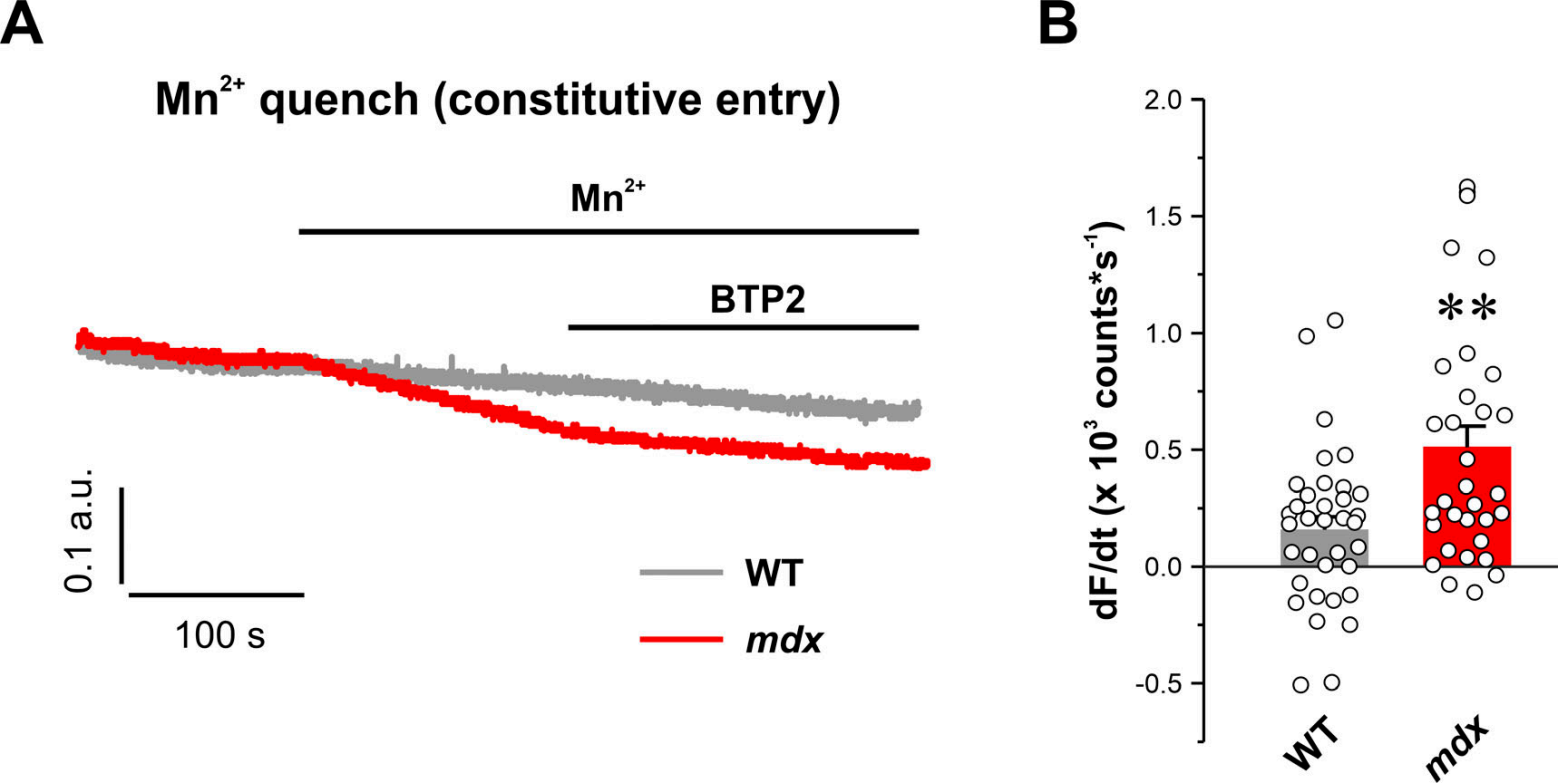
**Constitutive, BTP-2-sensitive Mn**^**2+**^ **quench. (A)** Representative superimposed traces of fura-2 fluorescence during application of 0.5 mM Mn^2+^ followed by application of 0.5 mM Mn^2+^ plus 10 μM BTP-2 in FDB fibers from WT (black trace, *n* = 36 fibers) and *mdx* (red trace, *n* = 32 fibers) mice in the absence of store depletion. **(B)** Quantitative analyses of the maximum rate of BTP-sensitive Mn^2+^ quench in the absence of store depletion. Data are shown as mean ± SEM; **, P = 0.00124, unpaired two-sample *t* test. Number of animals used: WT (*n* = 5) and *mdx* (*n* = 4).

**Figure S3.**
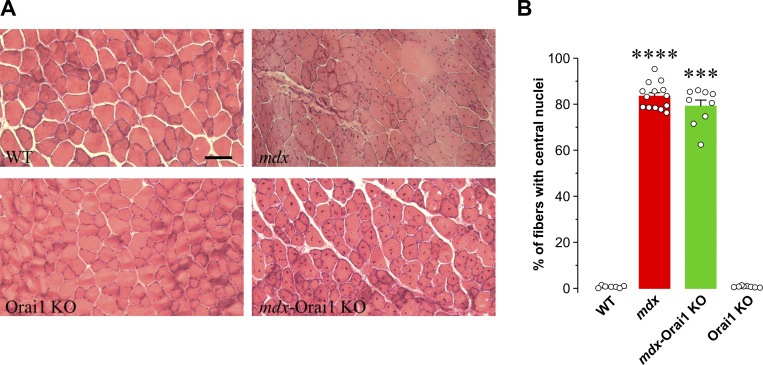
**Histological features in TA muscles. (A)** Representative histological sections of TA muscles stained with hematoxylin and eosin from WT (upper left), *mdx* (upper right), Orai1 KO (lower left), and *mdx*-Orai1 KO (lower right) mice. **(B)** Bar graph showing the percentage of TA fibers with central nuclei in WT (*n* = 9 TA sections), *mdx* (*n* = 14 TA sections), *mdx*-Orai1 KO (*n* = 9 TA sections), and Orai1 KO (*n* = 11 TA sections) mice. Data are shown as mean ± SEM. ****, P = 0.0000068; ***, P = 0.00020, relative to WT by one-way ANOVA followed by post-hoc Dunnett’s test. Number of animals used: WT (*n* = 3), *mdx* (*n* = 4), *mdx*-Orai1 KO (*n* = 3), and Orai1 KO (*n* = 3).

**Figure S4.**
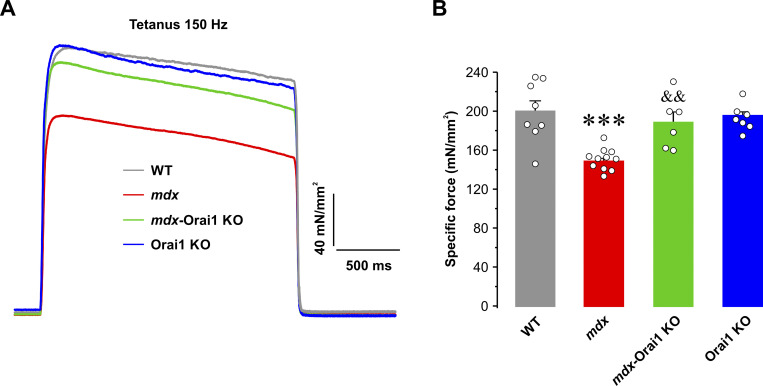
**Sustained tetanic force measurements in intact EDL muscles. (A)** Representative superimposed specific force (mN/mm^2^) traces elicited during a single 2-s, 150-Hz tetanic stimulation in EDL muscles from WT (gray; *n* = 8 muscles), *mdx* (red; *n* = 11 muscles), *mdx*-Orai1 KO (green; *n* = 6 muscles), and Orai1 KO (blue; *n* = 7 muscles) mice. **(B)** Bar graph summarizing average peak specific force in EDL muscles from the four genotypes shown in A. Data are shown as mean ± SEM. ***, P = 0.00018, relative to WT; ^&&^, P = 0.00863, relative to *mdx* by one-way ANOVA followed by post-hoc Tukey test. Number of mice used: WT (*n* = 5), *mdx* (*n* = 8), *mdx*-Orai1 KO (*n* = 4), and Orai1 KO (*n* = 5).

**Figure S5.**
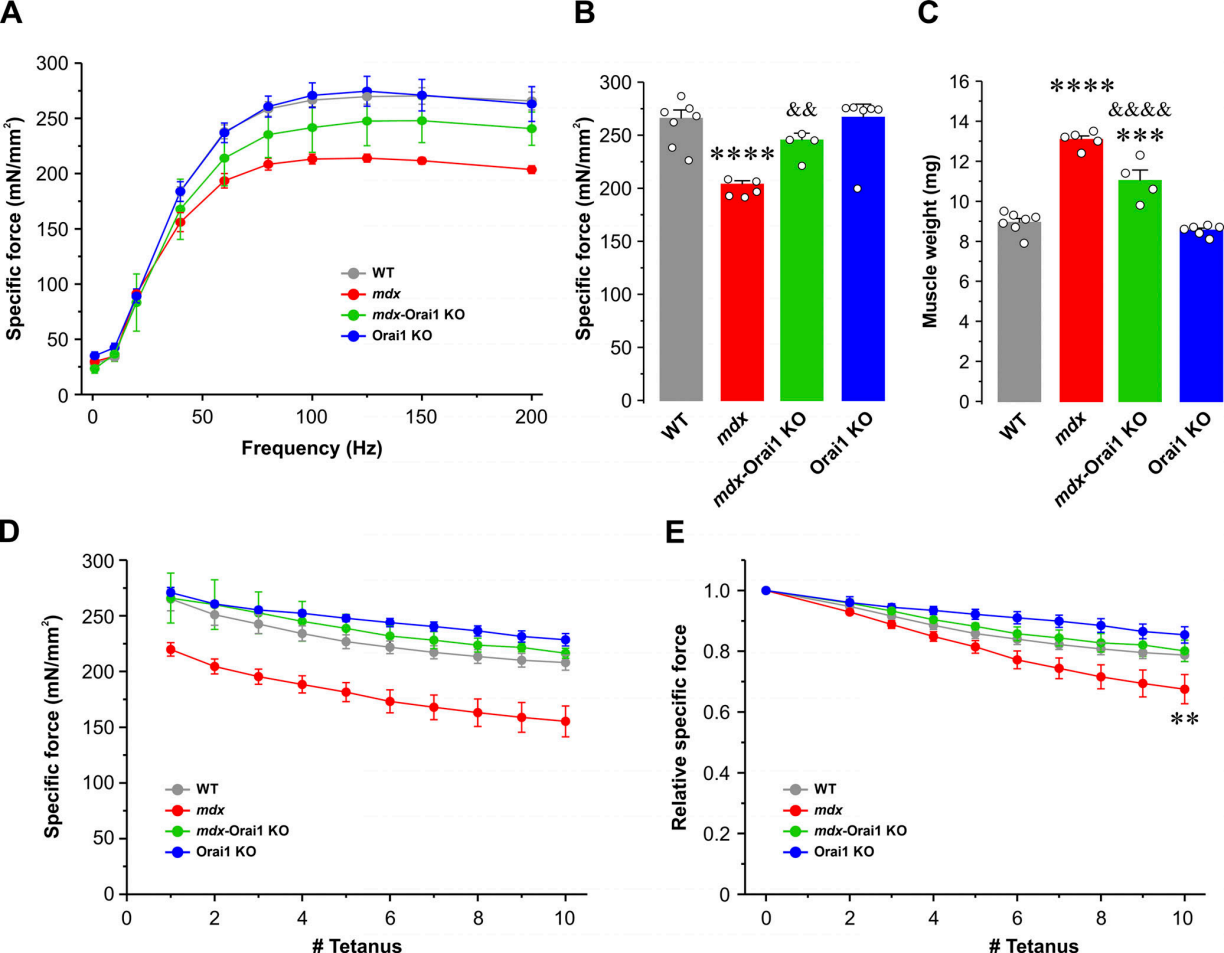
**Force–frequency relationship and eccentric contraction–induced damage in soleus muscles. (A)** Average specific force–frequency (1–200 Hz) relationship curves obtained in soleus muscles from WT (gray; *n* = 6 muscles), *mdx* (red; *n* = 9 muscles), *mdx*-Orai1 KO (green; *n* = 4 muscles), and Orai1 KO (blue; *n* = 6 muscles) mice. **(B)** Bar plot showing the average peak specific force measured at 200 Hz. **(C)** Bar graph summarizing average muscle weight for the soleus muscles shown in A and B. **(D and E)** Specific (D) and relative (E) force drop during 10 consecutive eccentric contractions. Each stimulation was 700 ms in duration, 500 ms of isometric contraction followed by 200 ms of eccentric contraction (muscles were subjected to a 10% stretch of the optimal length at 0.5 *L*_*o*_/s). Data are shown as mean ± SEM. In B, ****, P = 0.00061, relative to WT; ^&&^, P = 0.0062, relative to *mdx*. In C, ****, P = 0.000000012; ***, P = 0.00011, relative to WT; ^&&&&^, P = 0.0003, relative to *mdx*. In E, **, P = 0.0015, relative to WT by one-way ANOVA followed by post-hoc Tukey test. Number of mice used: WT (*n* = 3), *mdx* (*n* = 5), *mdx*-Orai1 KO (*n* = 3), and Orai1 KO (*n* = 4).

**Figure S6.**
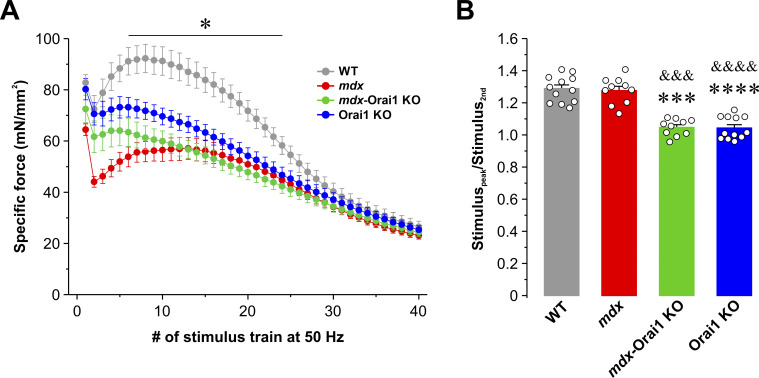
**Peak EDL-specific force during repetitive, high-frequency stimulation. (A)** Time course of peak specific force recorded in EDL muscles from WT (gray, *n* = 13 muscles), *mdx* (red, *n* = 10 muscles), *mdx*-Orai1 KO (green, *n* = 10 muscles), and Orai1 KO (blue, *n* = 12 muscles) mice during 40 consecutive, high-frequency stimulus trains (50 Hz, 500 ms, every 2.5 s). **(B)** Quantitative analyses of the peak fractional rebound increase in specific force following the second stimulus (Stimulus_peak_/Stimulus_2nd_) from the data shown in A. Data are shown as mean ± SEM; ***, P = 0.000000014; ****, P = 0.000000010 for *mdx*-Orai1 KO and Orai1 KO mice, respectively, relative to WT; ^&&&^, P = 0.000000075, ^&&&&^, P = 0.000000025 for *mdx*-Orai1 KO and Orai1 KO mice, respectively, relative to *mdx* by one-way ANOVA followed by post-hoc Tukey test. Number of animals used: WT (*n* = 8), *mdx* (*n* = 6), *mdx*-Orai1 KO (*n* = 5), and Orai1 KO (*n* = 7).

**Figure S7.**
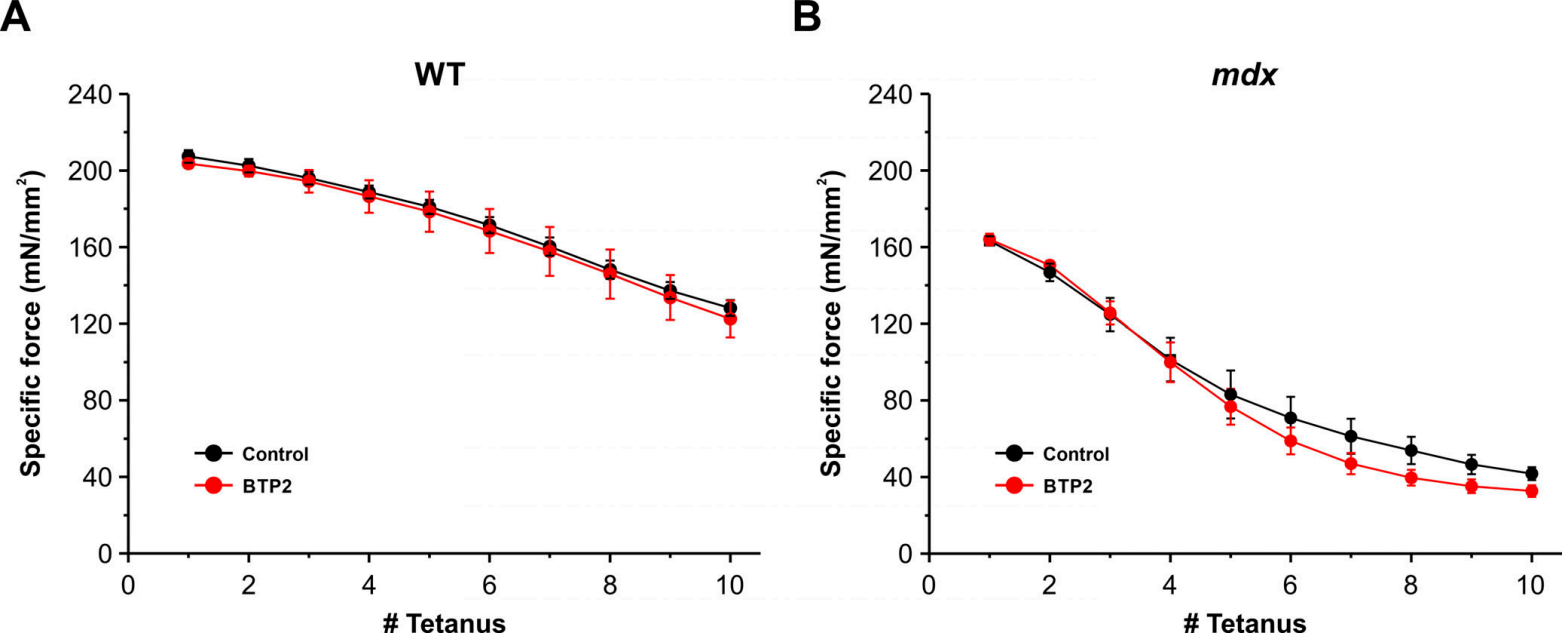
**Eccentric contraction–induced damage in EDL muscles in the absence or presence of BTP2.** **(A and B)** Specific force drop during 10 consecutive eccentric contractions in EDL muscles from WT (A, *n* = 4 muscles) and *mdx* (B, *n* = 4 muscles) mice while perfused in either control Ringer’s solution (black curve) or a Ringer’s solution supplemented with 10 µM BTP2 (red curves). Each stimulation was 700 ms in duration, 500 ms of isometric contraction followed by 200 ms of eccentric contraction (muscles were subjected to a 10% stretch of the optimal length at 0.5 *L*_*o*_/s). Data are shown as mean ± SEM by one-way ANOVA followed by post-hoc Tukey test. Number of mice used: WT (*n* = 3) and *mdx* (*n* = 3).

**Figure S8.**
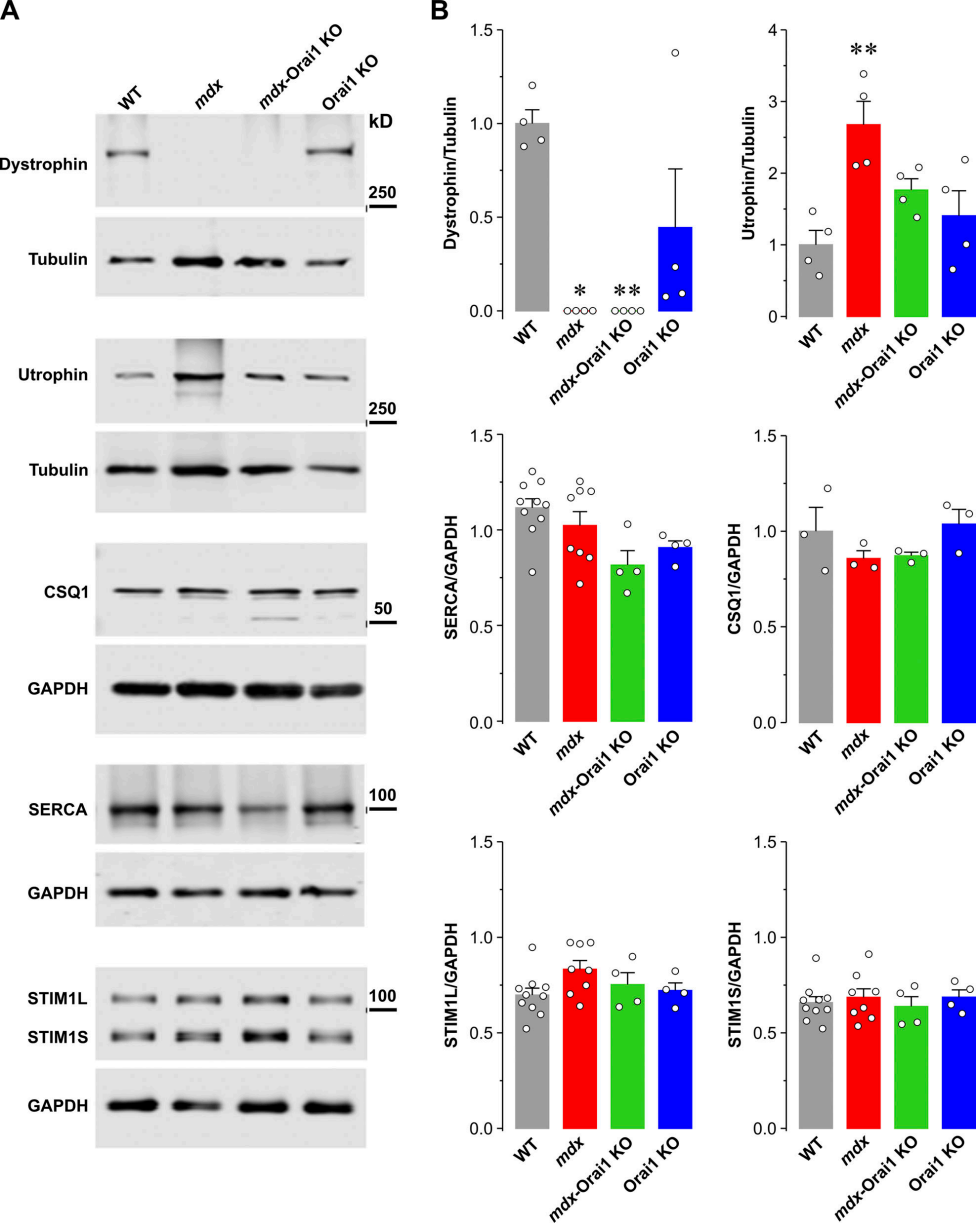
**Expression levels of proteins involved in Ca**^**2+**^ **homeostasis and maintaining sarcolemma integrity in EDL muscle homogenates. (A)** Representative immunoblots showing expression levels of dystrophin, utrophin, CSQ1, SERCA, and both the short (STIM1S) and long (STIM1L) STIM1 splice variants in EDL muscles from mice. **(B)** Bar graphs summarizing relative band intensities normalized to either β-tubulin (for dystrophin and utrophin) or GAPDH (for CSQ1, SERCA, STIM1L, and STIM1S). Number of mice used: WT (*n* = 4 mice), *mdx* (*n* = 4 mice), *mdx*-Orai1 KO (*n* = 4 mice), and Orai1 KO (*n* = 4 mice). Muscles from an additional six WT mice and four *mdx* mice were included for SERCA, STIM1S, and STIM1L. Data are shown as mean ± SEM. For dystrophin expression levels; *, P = 0.028 and **, P = 0.018 for *mdx* and *mdx*-Orai1 KO mice, respectively, relative to WT and for utrophin expression levels; **, P = 0.0024, relative to WT by one-way ANOVA followed by post-hoc Dunnett’s test. Source data are available for this figure: SourceData FS8.

Fig. S1 summarizes the impact of postdevelopmental, muscle-specific Orai1 ablation in *mdx* mice on grip strength and treadmill endurance. Fig. S2 compares the magnitude of BTP2-sensitive constitutive Ca^2+^ entry in FDB fibers from WT and *mdx* mice. Fig. S3 shows that postdevelopmental, muscle-specific Orai1 ablation does not reduce the high level of central nuclei observed in TA muscles from *mdx* mice. Fig. S4 presents the effect of postdevelopmental, muscle-specific Orai1 ablation in *mdx* mice peak EDL specific force during sustained tetanic stimulation. Fig. S5 summarizes the impact of postdevelopmental, muscle-specific Orai1 ablation in *mdx* mice on eccentric contraction–induced damage in soleus muscle. Fig. S6 shows the effect of postdevelopmental, muscle-specific Orai1 ablation in *mdx* mice on peak EDL specific force during repetitive, high-frequency stimulation. Fig. S7 shows that BTP2 exposure does not alter the magnitude of eccentric contraction–induced damage in EDL muscles from either WT or *mdx* mice. Fig. S8 summarizes the effect of postdevelopmental, muscle-specific Orai1 ablation in *mdx* mice on the expression levels of key proteins key involved membrane stability (dystrophin and utrophin) and SR Ca^2+^ levels (SERCA, CASQ1, STIM1L, and STIM1S).

## Results

### Postdevelopmental ablation of Orai1 in skeletal muscle of *mdx* mice

The dysregulation of Ca^2+^ homeostasis in the pathophysiology of DMD has been attributed to multiple mechanisms including microscopic membrane “tears,” as well as increased activity of Ca^2+^-permeable mechanosensitive and store-operated channels (Franco and Lansman, 1990; Fong et al., 1990; Vandebrouck et al., 2002; Millay et al., 2009; Goonasekera et al., 2014; Onopiuk et al., 2015). Previous works also found that muscle fibers from dystrophic mice exhibit increased expression levels of STIM1 and Orai1 that correlate with enhanced SOCE activity (Edwards et al., 2010; Zhao et al., 2012; Cully et al., 2012). To investigate the specific role of Orai1 in DMD, we crossed tamoxifen-inducible, muscle-specific Orai1 KO (Orai1 KO) male mice (Carrell et al., 2016) with *mdx* female mice homozygous for the dystrophin mutation. The resulting first generation of *mdx*-Orai1 heterozygous female mice were then bred with male Orai1 KO mice to obtain the four different genotypes used in this study (WT, *mdx*, *mdx*-Orai1 KO, and Orai1 KO mice; Fig. 1 A).

To assess the efficacy of postdevelopmental Orai1 ablation, we measured Orai1 transcript levels in TA muscle homogenates using both semiquantitative RT-PCR (Fig. 1 B) and quantitative RT-PCR approaches (Fig. 1 C). Similar to data reported previously (Zhao et al., 2012), Orai1 transcript levels, assessed with both approaches, were significantly increased (∼1.5-fold) in muscle of *mdx* mice compared with that of WT mice, while virtually absent (∼95% reduction in Orai1 mRNA level) in muscle from both *mdx*-Orai1 KO and Orai1 KO mice. We used the maximum rate of Mn^2+^ quench of fura-2 fluorescence in single FDB fibers following SR Ca^2+^ store depletion (pretreated for 1 h with 1 μM TG plus 15 μM CPA in a Ca^2+^-free Ringer’s solution) as an index of functional Orai1 expression and maximal SOCE in muscle as described previously (Wei-Lapierre et al., 2013). Consistent with the Orai1 transcript levels reported in Fig. 1, B and C, the maximum rate of Mn^2+^ quench of fura-2 fluorescence was ∼1.75 times greater in fibers from *mdx* mice (3.2 ± 0.8 counts/s × 10^3^) compared with that of WT mice (1.8 ± 0.8 counts/s × 10^3^), while essentially undetectable in fibers from both *mdx*-Orai1 KO and Orai1 KO mice (Fig. 1, D and E). Interestingly, postdevelopmental Orai1 ablation resulted in a partial rescue of reduced grip strength and increased fatigue during forced treadmill running (Fig. S1).

Prior studies reported increased constitutive Ca^2+^ entry (e.g., Ca^2+^ entry in the absence of active pharmacological store depletion) and resting Ca^2+^ levels in muscle fibers from *mdx* mice that are both reduced upon removal of extracellular Ca^2+^ (Altamirano et al., 2012). Thus, we addressed whether *mdx* fibers exhibited significant constitutive Orai1-dependent Ca^2+^ entry by quantifying Mn^2+^ quench of fura-2 fluorescence in WT and *mdx* fibers in the absence of actively depleting SR Ca^2+^ stores (naive fibers). To do this, Mn^2+^ quench of fura-2 fluorescence was measured in naive fibers first before and then after addition of BTP2, a potent SOCE channel inhibitor (Zitt et al., 2004). Results from these studies demonstrated that *mdx* fibers exhibited a significant constitutive BTP2-dependent Ca^2+^ entry that is not observed in WT fibers (Fig. S2).

### Postdevelopmental Orai1 ablation in skeletal muscle of *mdx* mice normalizes increased muscle fiber CSA and fibrosis

Increases in fiber size (hypertrophy), incidence of central nucleation (nuclei localized at the center of the fiber), inflammation, fibrosis, and necrosis are all hallmarks of muscular dystrophy (Verhaart and Aartsma-Rus, 2019; Deconinck and Dan, 2007). Thus, we assessed muscle fiber CSA in 10-μm-thick slices obtained from frozen TA muscles immunolabeled with anti-laminin antibody and nuclei stained with Hoechst dye (Fig. 2 A). As expected, average CSA of muscle fibers from *mdx* mice (2,126 ± 61 μm^2^) was approximately two times greater than that of fibers from WT mice (1,128 ± 21 μm^2^). Importantly, the CSA of fibers from *mdx*-Orai1 KO mice (1,214 ± 22 μm^2^) was significantly reduced compared with that observed for *mdx* mice (Fig. 2 B). We also measured hydroxyproline content, an index of muscle fibrosis, in diaphragm muscle homogenates from each of the four genotypes. While hydroxyproline content was markedly increased in diaphragm of *mdx* mice compared with that observed for both WT and Orai1 KO, postdevelopmental Orai1 ablation significantly reduced the increase in hydroxyproline content observed in *mdx* mice (Fig. 2 C). Finally, we evaluated the percentage of fibers from TA muscle that exhibit central nuclei, an index of muscle regeneration (Folker and Baylies, 2013). While virtually all muscle fibers from WT and Orai1 KO mice lacked centrally located nuclei, >80% of fibers from both *mdx* and *mdx*-Orai1 KO mice exhibited the presence of nuclei positioned at the center of the myofiber (Fig. S3). Thus, while postdevelopmental Orai1 ablation normalized muscle hypertrophy and hydroxyproline content in *mdx* mice, it did not mitigate a widely accepted biomarker of muscle regeneration (central nucleation).

### Postdevelopmental Orai1 ablation in skeletal muscle of *mdx* mice normalizes total releasable Ca^2+^ store content and resting myoplasmic Ca^2+^

**Figure 3.**
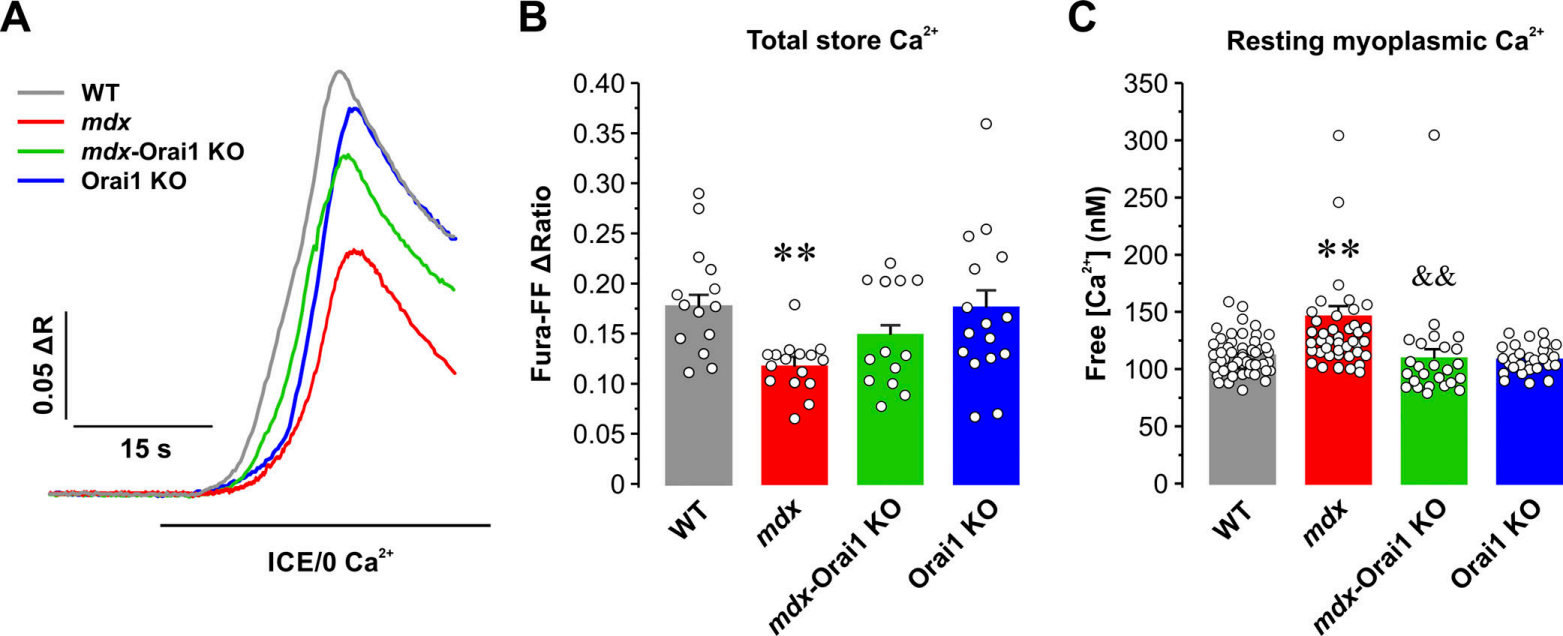
**Total releasable Ca**^**2+**^**-store content and resting myoplasmic free Ca**^**2+**^**concentration. (A)** Representative superimposed fura2-FF fluorescence traces in FDB fibers from WT (gray; *n* = 21 fibers), *mdx* (red; *n* = 23 fibers), *mdx*-Orai1 KO (green; *n* = 20 fibers), and Orai1 KO (blue; *n* = 23 fibers) mice before and during rapid local application of a Ca^2+^ store release cocktail (10 μM ionomycin, 30 µM CPA, and 100 μM EGTA; ICE/0 Ca^2+^). **(B)** Bar graph summarizing peak total releasable Ca^2+^ store content elicited during ICE application. **(C)** Quantitative analyses of resting myoplasmic free Ca^2+^ concentration (nM) recorded in fura-2–loaded FDB fibers from WT (*n* = 55 fibers), *mdx* (*n* = 43 fibers), *mdx*-Orai1 KO (*n* = 27 fibers), and Orai1 KO (*n* = 28 fibers) mice. Data are shown as mean ± SEM. In B, **, P = 0.0095, relative to WT; in C, **, P = 0.0014, relative to WT; ^&&^, P = 0.0038, relative to *mdx* by one-way ANOVA followed by post-hoc Tukey test. Number of mice used in B and C: WT (*n* = 3), *mdx* (*n* = 3), *mdx*-Orai1 KO (*n* = 3), and Orai1 KO (*n* = 3).

SOCE is a mechanism involved in the refilling of intracellular Ca^2+^ stores during repetitive, high-frequency stimulation that requires the presence of functional Orai1 (Wei-Lapierre et al., 2013; Boncompagni et al., 2017, Boncompagni et al., 2018; Sztretye et al., 2017; Michelucci et al., 2019; Michelucci et al., 2020). Therefore, we measured total releasable Ca^2+^ store content under resting conditions (e.g., without electrical stimulation) in single FDB fibers loaded with a low-affinity ratiometric Ca^2+^ dye, fura-FF, during application of a Ca^2+^ store release cocktail (ICE; 10 μM ionomycin, 30 μM CPA, and 100 μM EGTA). Total releasable Ca^2+^ store content in FDB fibers from *mdx* mice was significantly reduced (∼40%) compared with that observed in FDB fibers from WT mice, while being partially restored in fibers from *mdx*-Orai1 KO mice (Fig. 3, A and B). No difference in total releasable Ca^2+^ store content was observed between fibers from WT and Orai1 KO mice. Consistent with enhanced constitutive Ca^2+^ entry (Fig. S2) and SOCE (Fig. 1, D and E) in muscle fibers from *mdx* mice, we also observed a modest, but statistically significant, increase in resting myoplasmic free Ca^2+^ concentration (133 ± 5.6 nM) in FDB fibers from *mdx* mice compared with fibers from WT mice (111 ± 2.8 nM), which was normalized following postdevelopmental ablation of Orai1 in *mdx*-Orai1 KO mice (109 ± 8.2 nM; Fig. 3 C).

### Postdevelopmental Orai1 ablation in skeletal muscle of *mdx* mice improves electrically evoked Ca^2+^ release

**Figure 4.**
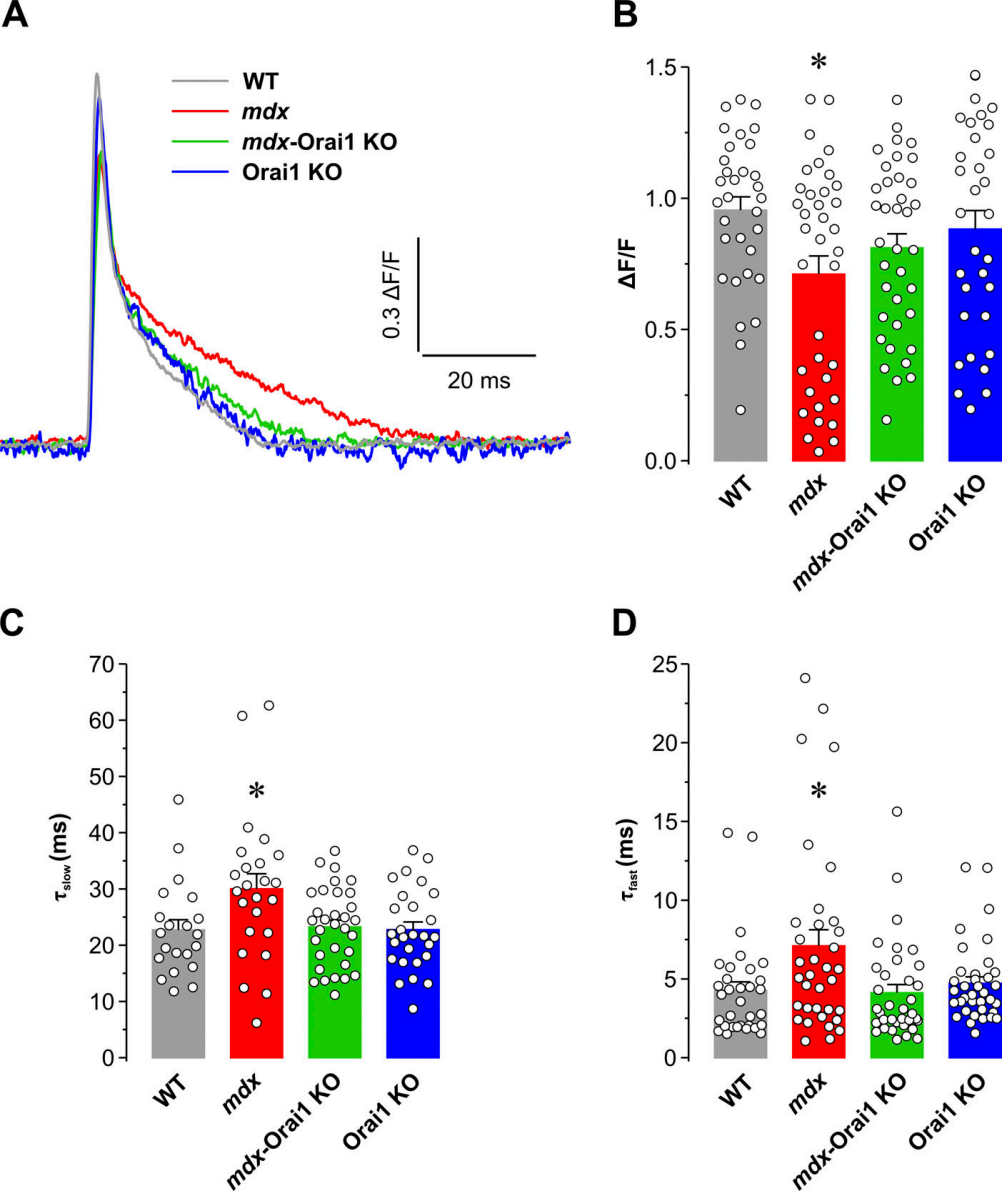
**Electrically evoked twitch Ca**^**2+**^ **transients. (A)** Representative superimposed traces of electrically evoked twitch Ca^2+^ transients (1 Hz) in mag-fluo-4–loaded FDB fibers from WT (gray; *n* = 32 fibers), *mdx* (red; *n* = 36 fibers), *mdx*-Orai1 KO (green; *n* = 43 fibers), and Orai1 KO (blue; *n* = 38 fibers) mice. **(B)** Bar graph summarizing peak Ca^2+^ transient amplitude elicited during twitch stimulation measured as the peak change in relative mag-fluo-4 fluorescence (∆*F*/*F*). **(C and D)** Bar graphs summarizing the fast (C) and slow (D) kinetic components of twitch Ca^2+^ transient decline obtained by fitting the decay phase with a second-order exponential function. Data are shown as mean ± SEM. In B, *, P = 0.0376; C, *, P = 0.0492; D, *, P = 0.0427, relative to WT by one-way ANOVA followed by post hoc Dunnett’s test. Number of mice used: WT (*n* = 3), *mdx* (*n* = 3), *mdx*-Orai1 KO (*n* = 4), and Orai1 KO (*n* = 3).

Previous studies reported that muscle fibers from *mdx* mice are characterized by a significant impairment of excitation–contraction coupling (Woods et al., 2004; Woods et al., 2005; Hollingworth et al., 2008; DiFranco et al., 2008; Capote et al., 2010), the process by which an action potential in the surface membrane is used to trigger intracellular Ca^2+^ release from the SR terminal cisternae at the triad junction, ultimately leading to muscle contraction. Therefore, we quantified the amplitude and kinetics of electrically evoked Ca^2+^ transients in FDB fibers during twitch (0.5-Hz) stimulation (Fig. 4). Results from these experiments revealed that peak Ca^2+^ transient amplitude in fibers from *mdx* mice was significantly reduced compared with that of fibers from WT mice, while Ca^2+^ transient amplitude returned to normal levels in fibers from *mdx*-Orai1 KO mice (Fig. 4, A and B). We fitted the decay phase of the Ca^2+^ transient to a second-order exponential equation, where the fast component of decay (*A*_*fast*_ and τ_*fast*_) primarily reflects Ca^2+^ binding to fast myoplasmic Ca^2+^ buffers, while the slow component of decay (*A*_*slow*_ and τ_*slow*_) is dominated by SERCA-mediated SR Ca^2+^ reuptake (Baylor and Hollingworth, 2003; Capote et al., 2005; Carroll et al., 1999). Results from these analyses revealed that both τ_*fast*_ (Fig. 4 C) and τ_*slow*_ (Fig. 4 D) were significantly greater in fibers from *mdx* mice compared with fibers from WT mice, while τ_*fast*_ and τ_*slow*_ in fibers from *mdx*-Orai1 KO and Orai1 KO mice were not significantly different from those of WT mice (Fig. 4, C and D). Thus, postdevelopmental ablation of Orai1 in *mdx* mice normalized Ca^2+^ transient amplitude and decay kinetics to values similar to those observed in WT mice.

### Postdevelopmental Orai1 ablation in skeletal muscle of *mdx* mice improves muscle-specific force production but enhances muscle fatigue during repetitive, high-frequency stimulation

**Figure 5.**
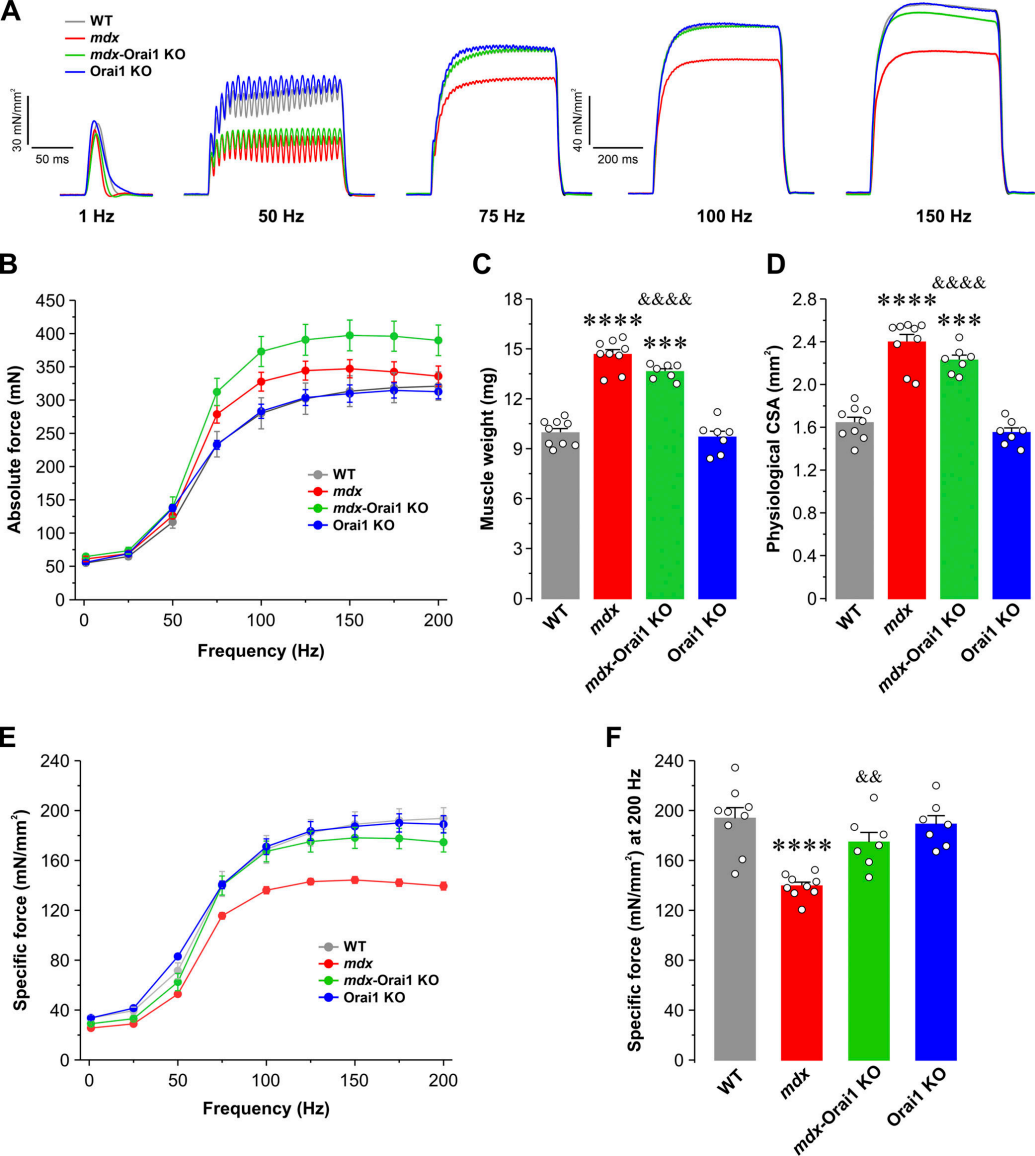
**EDL-specific force–frequency relationship. (A)** Representative superimposed specific force traces elicited at different stimulation frequencies (1, 50, 75, 100, and 150 Hz) in EDL muscles from WT (gray; *n* = 9 muscles), *mdx* (red; *n* = 7 muscles), *mdx*-Orai1 KO (green; *n* = 8 muscles), and Orai1 KO (blue; *n* = 8 muscles) mice. **(B)** Average absolute force–frequency relationship curves for each of the four genotypes shown in A. **(C and D)** Bar graphs showing average muscle weight (C) and physiological CSA (D) for the EDL muscles shown in B. **(E)** Average specific force–frequency relationship curves for each of the four genotypes shown in A. **(F)** Bar plot showing the average peak specific force measured at 200 Hz. Data are shown as mean ± SEM. In C, ****, P = 0.000000091; ***, P = 0.00000098 for *mdx* and *mdx*-Orai1 KO, respectively, relative to WT; ^&&&&^, P = 0.0000082, relative to *mdx*. In D, ****, P = 0.00000011; ***, P = 0.00019 for *mdx* and *mdx*-Orai1 KO, respectively, relative to WT; ^&&&&^, P = 0.00097, relative to *mdx*. F; ****, P = 0.000013, relative to WT; ^&&^, P = 0.0064 relative to *mdx* by one-way ANOVA followed by post-hoc Tukey test. Number of mice used: WT (*n* = 4), *mdx* (*n* = 5), *mdx*-Orai1 KO (*n* = 4), and Orai1 KO (*n* = 5).

A marked reduction in the ability to generate force is a hallmark feature of DMD (Lynch et al., 2001). Therefore, we determined the effect of postdevelopmental Orai1 ablation on specific force production in intact EDL muscles. Excised EDL muscles of all genotypes were subjected to a force–frequency protocol with frequencies ranging from 1 to 250 Hz (Fig. 5 A). Results from these experiments revealed that absolute force (mN) was greater for EDL muscles from both *mdx* and *mdx*-Orai1 KO mice compared with that observed for EDL muscles from either WT or Orai1 KO mice (Fig. 5 B). This finding was consistent with the significantly larger muscle mass (Fig. 5 C) and physiological CSA (Fig. 5 D) of EDL muscles from 13–16-wk-old *mdx* mice. However, EDL muscles from *mdx* mice exhibited a reduction of peak specific force (mN/mm^2^) for all frequencies ≥50 Hz (Fig. 5 E). Specifically, peak EDL specific force at 200 Hz was 193.7 ± 8.6 mN/mm^2^ for WT mice, but only 139.4 ± 3.1 mN/mm^2^ for EDL muscles from *mdx* mice (Fig. 5 F). EDL muscles from *mdx-*Orai1 KO mice exhibited peak specific force at 200 Hz that was not significantly different from that of WT mice (174.6 ± 7.8 mN/mm^2^). These results were confirmed during application of a 2-s tetanus at 150 Hz, which showed that postdevelopmental ablation of Orai1 normalized the marked reduction in peak specific force observed in EDL muscles from *mdx* mice (Fig. S4). Similar results were observed in soleus muscles (Fig. S5, A and B).

Orai1 ablation leads to increased muscle fatigue during repetitive, high-frequency stimulation (Wei-Lapierre et al., 2013; Boncompagni et al., 2017; Michelucci et al., 2019, Michelucci et al., 2020). To directly assess the impact of postdevelopmental Orai1 ablation on muscle fatigue during prolonged activity, we subjected excised EDL muscles to a standard repetitive, high-frequency stimulation paradigm (40 stimulus trains of 50 Hz and 500 ms in duration delivered every 2.5 s). EDL muscles from WT mice exhibited an initial drop in specific force during the second stimulus train followed by a rebound increase (∼30%) in specific force production (Fig. S6, A and B) similar to that previously reported to be associated with SOCE activity (Michelucci et al., 2019; Michelucci et al., 2020). While EDL muscles from *mdx* mice exhibited reduced specific force production during the first 50-Hz stimulation (consistent with results in Fig. 5 E), EDL muscles from *mdx* mice nevertheless also exhibited an ∼30% rebound increase in specific force after the second stimulus (Fig. S6, A and B). Consistent with prior findings (Michelucci et al., 2019), Orai1 ablation abolished this rebound increase in specific force production after the second stimulus in both *mdx*-Orai1 KO and Orai1 KO mice.

### Postdevelopmental Orai1 ablation in skeletal muscle of *mdx* mice reduces eccentric contraction–induced muscle damage

**Figure 6.**
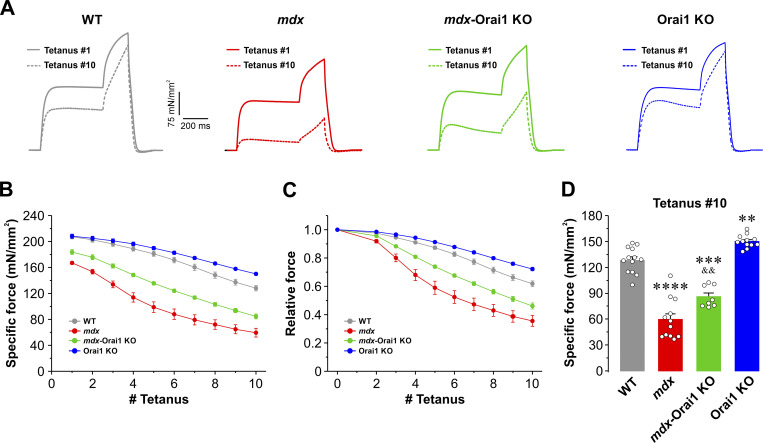
**Eccentric contraction–induced damage in EDL muscles. (A)** Representative superimposed specific force traces during the 1st (continuous line) and 10th (dashed line) tetanic eccentric contraction (700 ms, 150 Hz, every 120 s) in EDL muscles from WT (gray; *n* = 13 muscles), *mdx* (red; *n* = 12 muscles), *mdx*-Orai1 KO (green; *n* = 10 muscles), and Orai1 KO (blue; *n* = 12 muscles) mice. Each stimulation was 700 ms in duration, 500 ms of isometric contraction followed by 200 ms of eccentric contraction (muscles subjected to a 10% stretch of the optimal length at 0.5 *L*_*o*_/s). **(B and C)** Specific (B) and relative (C) force drop during 10 consecutive eccentric contractions. **(D)** Bar graph showing peak specific force measured during tetanus #10. Data are shown as mean ± SEM. ****, P = 0.0000006; ***, P = 0.000003; **, P = 0.00671 for *mdx*, *mdx*-Orai1 KO, and Orai1 KO mice, respectively, relative to WT; ^&&^, P = 0.00394, relative to *mdx*, one-way ANOVA followed by the post-hoc Tukey test. Number of mice used: WT (*n* = 7), *mdx* (*n* = 7)*, mdx*-Orai1 KO (*n* = 5), and Orai1 KO (*n* = 6).

Besides a reduction in isometric force generation, dystrophic muscles are also characterized by an increased susceptibility to eccentric contraction–induced damage (Dellorusso et al., 2001; Liu et al., 2005; Whitehead et al., 2008). To evaluate susceptibility to damage, a different set of EDL muscles were subjected to a previously validated ex vivo eccentric contraction damage protocol (Lindsay et al., 2020) consisting of 10 consecutive, 700-ms stretching tetani delivered at 150 Hz (500 ms of isometric contraction followed by a 200 ms 10% stretch of *L*_*o*_ at a speed of 0.5 *L*_*o*_/s) applied every 120 s (Fig. 6 A). The ability to develop force following subsequent eccentric contractions declined modestly in EDL muscles from WT mice (61.8 ± 1.9% of initial specific force during the 10th eccentric contraction). In contrast, the eccentric contraction–induced force drop was considerably more pronounced in EDL muscles from *mdx* mice (35.5 ± 3.8% of initial specific force during the 10th eccentric contraction), consistent with a higher susceptibility to mechanical stress-induced damage (Fig. 6, B–D). Postdevelopmental ablation of Orai1 significantly reduced eccentric contraction–induced force drop in EDL muscles from *mdx* mice (46.1 ± 2.6% of residual force at the 10th eccentric contraction). Interestingly, postdevelopmental KO of Orai1 also significantly reduced eccentric contraction–induced force drop compared with that observed in EDL muscles from WT mice (72.2 ± 1.2% of initial force during the 10th eccentric contraction). Similar results were also obtained in soleus muscles (Fig. S5, C and D).

To determine if muscle damage is augmented by Orai1-dependent SOCE during each eccentric contraction, we performed experiments on intact EDL muscles from WT and *mdx* mice subjected to the same eccentric contraction protocol conducted in the presence of either control Ringer’s solution or Ringer’s solution supplemented with 10 μM BTP2. Results from these experiments revealed that eccentric contraction–induced damage was unaltered by acute block of SOCE with BTP2 in EDL muscles from either WT (Fig. S7 A) or *mdx* (Fig. S7 B) mice. Together, these studies suggest that chronic inhibition of Orai1-dependent SOCE is required to protect skeletal muscle from eccentric contraction–induced injury.

## Discussion

### Main findings of the study

In this study, we report that enhanced Orai1-dependent Ca^2+^ entry contributes to the pathogenesis of DMD. Consistent with prior reports (Edwards et al., 2010; Zhao et al., 2012), skeletal muscle from *mdx* mice exhibits increased Orai1 transcript levels and enhanced constitutive Ca^2+^ entry and SOCE (Fig. 1 and Fig. S2), which correlated with (1) reduced total releasable Ca^2+^ store content and increased myoplasmic free Ca^2+^ concentration (Fig. 2); (2) increased myofiber CSA, fibrosis, and central nucleation (Fig. 3); (3) reduced peak electrically evoked Ca^2+^ transient amplitude and prolonged Ca^2+^ transient decay (Fig. 4); (4) reduced specific force production (Fig. 5), and (5) increased susceptibility to eccentric contraction–induced damage (Fig. 6). Collectively, this constellation of functional and histopathological alterations represents the dystrophic phenotype of *mdx* mice.

Importantly, we found that postdevelopmental, muscle-specific KO of Orai1 significantly ameliorates the dystrophic phenotype of young adult (3–4-mo-old) *mdx* mice. Specifically, ablation of Orai1-dependent Ca^2+^ entry in adult *mdx*-Orai1 KO mice partially restores total releasable Ca^2+^ store content and normalizes the resting myoplasmic free Ca^2+^ concentration to levels similar to that of WT. Furthermore, both the peak amplitude and slowed decay phase of electrically evoked Ca^2+^ transients in single FDB fibers, as well as peak specific force production in excised EDL and soleus muscles, are significantly improved. However, consistent with that reported previously in WT mice (Michelucci et al., 2019), postdevelopmental ablation of Orai1 in *mdx* mice also results in a reduced ability to maintain force production during repetitive high-frequency stimulation (Fig. S6).

Finally, postdevelopmental Orai1 KO also reduces myofiber hypertrophy and fibrosis, as well as protects muscles from eccentric contraction–induced muscle damage. However, Orai1 KO does not alter the percentage of centrally nucleated fibers, consistent with ongoing muscle regeneration even in the absence of Orai1 expression. Overall, these results extend prior findings (Goonasekera et al., 2014) by demonstrating that postdevelopmental functional Orai1 deficiency reduces the severity of muscular dystrophy in *mdx* mice through normalization of both Ca^2+^ homeostasis and sarcolemmal integrity.

### Postdevelopment Orai1 KO normalizes Ca^2+^ handling in *mdx* muscle

A long-standing observation in the field is that Ca^2+^ homeostasis is disrupted in muscular dystrophy. Several interrelated mechanisms have been proposed to contribute to altered Ca^2+^ homeostasis in muscular dystrophy including (1) enhanced trans-sarcolemmal Ca^2+^ entry (Michelucci et al., 2018; Zabłocka et al., 2021; Mareedu et al., 2021), (2) increased RyR1 Ca^2+^ leak (Bellinger et al., 2009; Andersson et al., 2012), (3) impaired SR Ca^2+^ reuptake (Viner et al., 1996; Sharov et al., 2006; Dremina et al., 2007), (4) increased oxidative stress (Whitehead et al., 2008; Petrillo et al., 2017), and (5) mitochondrial dysfunction (Mareedu et al., 2021; Budzinska et al., 2021). Indeed, it is likely that multiple mechanisms work in concert, and possibly at different levels in different muscles, to contribute to altered Ca^2+^ homeostasis in muscular dystrophy.

Although several areas of controversy remain, there is a general consensus that Ca^2+^ homeostatic mechanisms are altered and intracellular free Ca^2+^ concentration is modestly increased in dystrophic muscles. Different mechanisms are proposed to contribute to altered Ca^2+^ homeostasis including excessive Ca^2+^ entry (e.g., microtears, mechanosensitive channels, TrpC channels, and SOCE channels). Prior studies demonstrated a clear relationship between increases in sarcolemmal Ca^2+^ entry and resting intracellular Ca^2+^ concentration in muscle fibers from *mdx* mice and other genetic models of muscular dystrophy (Yeung et al., 2005; Altamirano et al., 2012). In one of the most comprehensive studies in this regard, Altamirano et al. (2012) used double-barrel Ca^2+^ electrode measurements to quantify subsarcolemmal free Ca^2+^ concentration in fibers from WT and *mdx* mice under different conditions designed to inhibit extracellular Ca^2+^ entry (e.g., low Ca^2+^, 0 Ca^2+^, 20 μM Gd^3+^, 0 Ca^2+^ + 20 μM Gd^3+^). Consistent with prior findings (Mallouk et al., 2000), this study confirmed that subsarcolemmal Ca^2+^ is elevated in muscle fibers from *mdx* mice and that this increase is at least partially reduced by removing extracellular Ca^2+^ and/or blocking Ca^2+^ entry channels with Gd^3+^. Our findings further indicate that this increased constitutive Ca^2+^ entry observed in fibers from *mdx* mice is blocked by BTP2 (Fig. S2).

By altering net trans-sarcolemmal Ca^2+^ influx/efflux, enhanced Ca^2+^ entry via an Orai1-dependent pathway could contribute to the increase in steady-state myoplasmic free Ca^2+^ concentration observed in fibers from *mdx* mice, consistent with the cell boundary theorem (Friel and Tsien, 1992; Ríos, 2010). The cell boundary theorem states that a change in steady-state resting free Ca^2+^ concentration requires a net change in Ca^2+^ influx/efflux across the surface membrane. We found that muscle fibers from *mdx* mice exhibit a constitutive BTP2-sensitive Ca^2+^ influx. Assuming that Ca^2+^ efflux mechanisms are unaltered in fibers from *mdx* mice as suggested previously (Cully et al., 2012), an increase in net Ca^2+^ influx would be expected to result in an increase in steady-state resting free Ca^2+^ concentration. A limitation of our study is that we are not able to provide a more quantitative description, since we did not directly measure net Ca^2+^ influx (only Mn^2+^ influx) and efflux rates in fibers from WT and *mdx* mice.

In addition to increases in both trans-sarcolemmal Ca^2+^ influx and steady-state resting Ca^2+^ levels, acute or dynamic changes in intracellular Ca^2+^ concentration also depend on (1) myoplasmic Ca^2+^ binding proteins (e.g., parvalbumin), (2) SR Ca^2+^ reuptake (e.g., SERCA), and (3) SR Ca^2+^ leak/release via RyR1. Interestingly, a reduction of SERCA activity (Kargacin and Kargacin, 1996; Divet and Huchet-Cadiou, 2002; Divet et al., 2005; see also Fig. 4) is observed in skeletal muscle of *mdx* mice. Impaired SR Ca^2+^ reuptake due to reduced SERCA expression and/or activity could at least in part explain the reduction in total releasable Ca^2+^ store content (Fig. 3 B) and slowed Ca^2+^ transient decline (Fig. 4, C and D) observed in FDB fibers from *mdx* mice. The slower rate of Ca^2+^ transient decline in fibers from *mdx* mice is in line with results reported previously. For instance, Goonasekera et al. (2014) reported a similar slowed rate of Ca^2+^ transient decay in FDB fibers from δ-sarcoglycan–null (*Sgcd*^−/−^) mice (Goonasekera et al., 2014). In addition, prior studies reported a significant reduction in SERCA function in EDL muscle of *mdx* mice (Kargacin and Kargacin, 1996; Divet and Huchet-Cadiou, 2002; Divet et al., 2005; Gehrig et al., 2012). In line with this idea, muscle-specific overexpression of SERCA1 restores both peak Ca^2+^ transient amplitude and decay kinetics, as well as dramatically attenuates the dystrophic phenotype in two different muscular dystrophy mouse models (*mdx* and *Sgcd*^−/−^ mice; Goonasekera et al., 2011).

Importantly, we found that postdevelopmental ablation of Orai1 similarly restores key functional measures of SERCA activity in FDB fibers including total releasable Ca^2+^ store content (Fig. 3) and Ca^2+^ transient amplitude/decay (Fig. 4). While the changes in the rate of Ca^2+^ transient decay observed in *mdx* mice and restoration after Orai1 KO could be explained by deceased SERCA1 expression in muscle of *mdx* mice that is normalized following Orai1 ablation, we did not find any statistically significant differences in SERCA1 expression across any of the four genotypes (Fig. S8 B). Thus, the precise mechanism by which Orai1 ablation normalizes these functional measures of SERCA activity without altering SERCA expression remains unclear. As one possibility, Orai1 ablation could reduce the inhibitory effects of oxidative/nitrosative modifications on SERCA function reported previously (Viner et al., 1996; Sharov et al., 2006; Dremina et al., 2007; Qaisar et al., 2021). Another possible explanation is that loss of Orai1 could either reduce expression of an endogenous SERCA inhibitor (e.g., sarcolipin or myoregulin; Schneider et al., 2013; Chambers et al., 2022) or increase expression of an endogenous SERCA activator (e.g., DWORF; Nelson et al., 2016). Further studies will be required to evaluate the role of these potential mechanisms.

The reduction in total releasable Ca^2+^ store content likely contributes to the reduction in the amplitude of electrically evoked Ca^2+^ release observed in our study and also reported by others (Head, 2010; Hollingworth et al., 2008; Woods et al., 2004). In addition to the contribution of SERCA pumps, total releasable Ca^2+^ store content also depends on the Ca^2+^ buffering capacity of the SR, which in fast-twitch muscle is largely dependent on the expression of CSQ1 (Michelucci et al., 2020). While some studies reported a significant decrease in CSQ content in young (∼8-wk-old) *mdx* muscles (Doran et al., 2004; Pertille et al., 2010), this is not typically observed at the older age (13–16 wk old) used in this study (Culligan et al., 2002; Ferretti et al., 2009; Matsumura et al., 2013; Barros Maranhão et al., 2015). Consistent with this, we did not find significant differences in CSQ1 expression in Western blot analyses of EDL muscle homogenates from 3–4-mo-old mice across all four genotypes (Fig. S8). However, the potential impact of postdevelopmental Orai1 ablation on the expression level of other lower-abundance SR Ca^2+^ binding proteins in muscle (e.g., histidine-rich Ca^2+^ binding protein, junctate, sarcolumenin, and CSQ-like binding proteins; Rossi and Dirksen, 2006; Culligan et al., 2002) remains to be determined.

In addition to decreased SERCA activity, the observed reduction in releasable Ca^2+^ store content could be due in part to increased RyR1 Ca^2+^ leak. Bellinger et al. (2009) demonstrated that RyR1 isolated from *mdx* skeletal muscle exhibits posttranslational modifications (e.g., RyR1-nitrosylation) that result in reduced binding of the regulatory protein FKBP12, with a consequent increase of RyR1 opening and Ca^2+^ leak. The leak of Ca^2+^ through oxidized RyR1 channels (together with reduced SERCA activity and potentially reduced SR Ca^2+^ buffering) would be expected to lead to a reduction in releasable SR Ca^2+^ store content. Consistent with this, treatment for 6 wk with an antioxidant (*N*-acetylcysteine) significantly reduced the dystrophic phenotype of *mdx* mice (Whitehead et al., 2008). A similar pathogenic role for altered Ca^2+^ handling and excessive ROS production was demonstrated for other muscle disorders (malignant hyperthermia and central core disease) characterized by enhanced nitrosative/oxidative stress, RyR1 nitrosylation, SR Ca^2+^ leak, and altered Ca^2+^ homeostasis (Durham et al., 2008; Lanner et al., 2012; Michelucci et al., 2015; Michelucci et al., 2017a; Michelucci et al., 2017b; Michelucci et al., 2021). However, whether functionally relevant ROS/RNS-dependent posttranslational modifications to proteins of the SOCE machinery (STIM1 and Orai1) occur in dystrophic muscle is unknown and certainly warrants future investigation.

### Postdevelopmental Orai1 KO limits membrane damage during eccentric contraction

An unexpected finding of this study was the protective effect of postdevelopmental Orai1 ablation on the susceptibility of muscle to eccentric contraction–induced damage. Specifically, Orai1 KO in EDL and soleus muscles in both WT (Orai1-KO) and *mdx* (*mdx*-Orai1 KO) mice exhibit a significantly reduced force drop following a series of eccentric contractions. Interestingly, this reduction in muscle damage was independent of acute Orai1-dependent Ca^2+^ entry, since the eccentric contraction–induced force drop was unaffected by exposure to 10 μM BTP2, a validated inhibitor of SOCE in muscle (Wei-LaPierre et al., 2022). These results suggest that the enhanced protection from eccentric contraction–induced damage does not result from acute Orai1-dependent Ca^2+^ entry, but rather from long-term effects of the loss of Orai1-dependent Ca^2+^ entry (e.g., reduced activation of calpains or other Ca^2+^ dependent processes). Indeed, uncontrolled protein degradation promoted by Ca^2+^-activated calpains is recognized as a key pathophysiological aspect of muscular dystrophy (Spencer et al., 1995; Tidball and Spencer, 2002; Spencer and Mellgren, 2002). Alternatively, a potential scaffolding role of the Orai1 complex in membrane integrity cannot be excluded.

Zhao et al. (2012) reported that chronic (2 wk) in vivo inhibition of SOCE with BTP2 significantly reduces both the rate of proteolytic events mediated by calpains and the progression of the dystrophic phenotype of *mdx* mice. In an analogous manner, early developmental (or constitutive) inhibition of SOCE in muscle following muscle-specific expression of dominant-negative Orai1 (dnOrai1) also mitigated the dystrophic phenotype in both *mdx* mice and *Sgcd*^*−/−*^ mice (Goonasekera et al., 2014). However, BTP2 and dnOrai1 inhibit both Orai1 and TrpC channels, and constitutive muscle-specific expression of dnOrai1 results in several additional important developmental effects in muscle (e.g., reduced type 1 fiber content and CSA; Wei-Lapierre et al., 2013). Our results show that specifically targeting Orai1 in fully developed muscle of adult mice is sufficient to mitigate the dystrophic phenotype of *mdx* mice. These results provide evidence that inhibition of Orai1 in DMD patients may provide some level of protection or slow disease progression even after disease onset. However, the important role of Orai1 in mitigating muscle fatigue during repetitive stimulation (Michelucci et al., 2019; Fig. S6) may limit the therapeutic benefit of inhibiting Orai1 function in individuals with DMD.

We also investigated if the protective effect of postdevelopmental Orai1 ablation could be explained by compensatory changes in the expression of utrophin, a structural and functional paralogue of dystrophin (Ibraghimov-Beskrovnaya et al., 1992). Utrophin localizes at the neuromuscular junction of normal adult fibers (Love et al., 1989; Tinsley et al., 1992), while also being abundantly present throughout the sarcolemma during muscle development and damage-induced muscle regeneration (Ohlendieck et al., 1991; Amenta et al., 2011). Utrophin is also upregulated and localized along the sarcolemma in regenerating fibers of patients with muscular dystrophy (Helliwell et al., 1992), and transgenic overexpression of utrophin suppresses the dystrophic phenotype of *mdx* mice (Tinsley et al., 1998; Fisher et al., 2001). However, our results indicate that the reduced eccentric contraction–induced force drop observed following postdevelopmental Orai1 ablation occurs in the absence of a detectable change in utrophin expression (Fig. S8).

### Conclusions

The findings reported in this study provide new insights regarding the role of Orai1 in DMD pathogenesis. Collectively, our results support the hypothesis that increased Orai1-dependent SOCE potentiates a cascade of downstream pathways that ultimately culminate in increased myofiber fragility and death. An important implication of these studies is that mitigation of the dystrophic phenotype can be achieved by targeting these Orai1-dependent pathways even after muscle development is complete. Nevertheless, several unresolved issues remain to be addressed. For instance, whether other Orai isoforms (Orai2 and Orai3) are involved in the DMD phenotype remains an open and unsolved issue. While we did not directly assess potential compensatory changes in Orai2 and/or Orai3 expression in muscle of Orai1 KO and *mdx*-Orai1 KO mice, the lack of detectable store-dependent Mn^2+^ quench in fibers from Orai1 KO and *mdx*-Orai1 KO mice (Fig. 1 E) argues against a compensatory upregulation of alternative Orai1-independent Ca^2+^ influx mechanisms. However, a comprehensive assessment of this possibility will require further study. Finally, future studies are needed to elucidate the precise downstream mechanisms responsible for how enhanced Orai1-mediated SOCE potentiates the dystrophic phenotype, as well as to determine if therapeutic strategies designed to target these mechanisms can be effectively translated to the treatment of children with DMD.

## Supplementary Material

SourceData FS8 is the source file for Fig. S8.
