# Supplementary material for: Postdevelopmental knockout of Orai1 improves muscle pathology in a mouse model of Duchenne muscular dystrophy
Source: J Gen Physiol. 2022 Aug 8;154(9):e202213081. doi: 10.1085/jgp.202213081 (PMC9365874; doi:10.1085/jgp.202213081)
Supplement: SourceData FS8 — is the source file for Fig. S8. [file JGP_202213081_SourceDataFS8.pdf]

Dystrophin

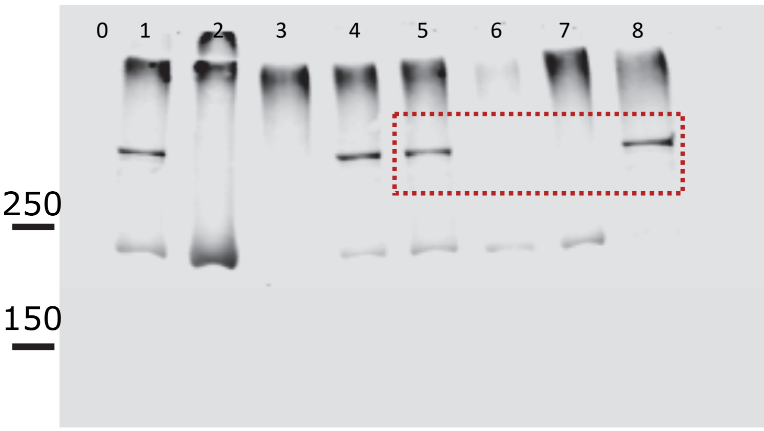

Tubulin

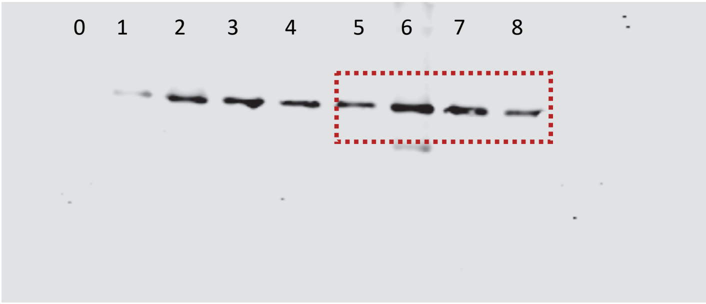

Utrophin

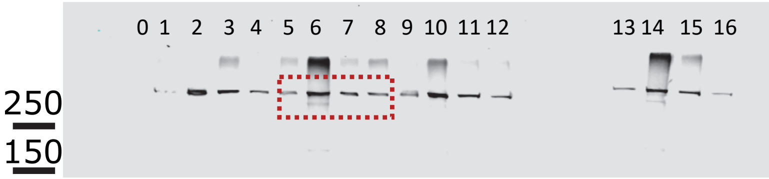

Tubulin

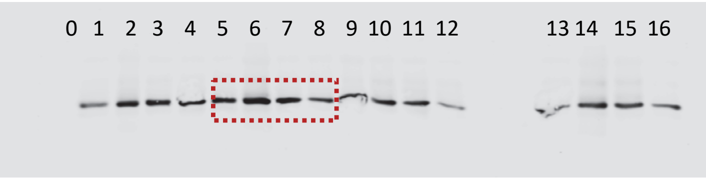

CSQ1 & GAPDH

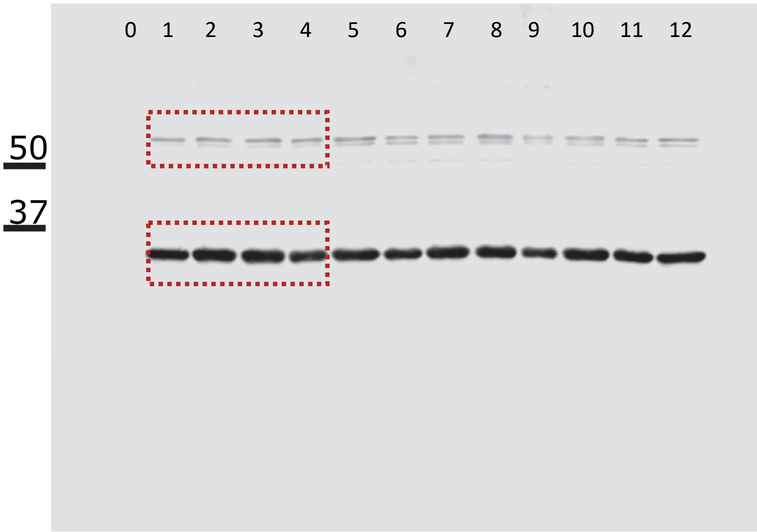

SERCA & GAPDH

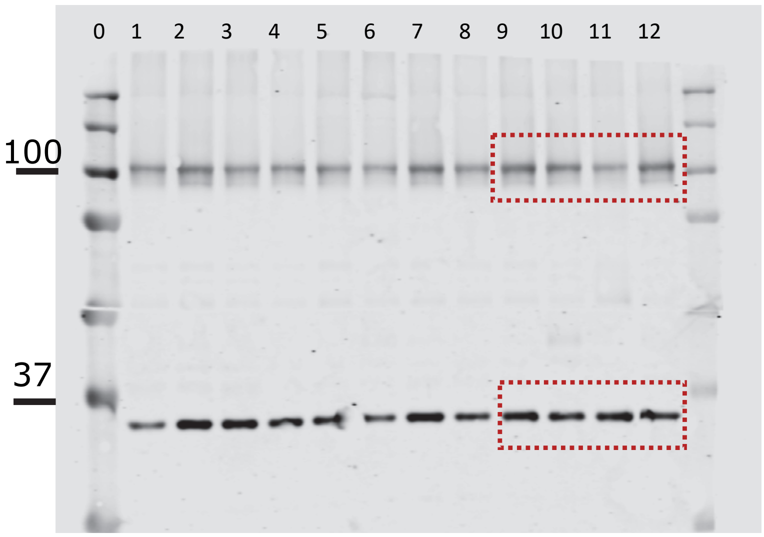

STIM1L, STIM1S & GAPDH

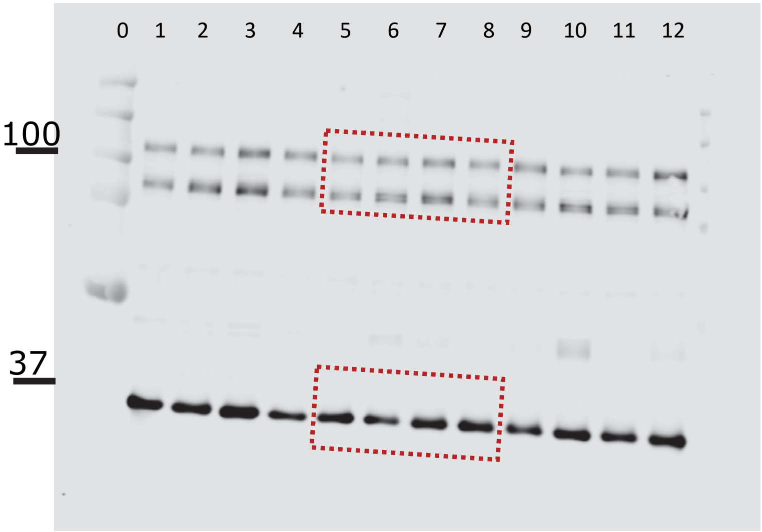

1. WT
2. *mdx*
3. *mdx*-Orai1 KO
4. Orai1 KO
5. WT
6. *mdx*
7. *mdx*-Orai1 KO
8. Orai1 KO
9. WT
10. *mdx*
11. *mdx*-Orai1 KO
12. Orai1 KO
13. WT
14. *mdx*
15. *mdx*-Orai1 KO
16. Orai1 KO
